# Evaluating the significance of contact maps in low-homology protein modeling using contact-assisted threading

**DOI:** 10.1038/s41598-020-59834-2

**Published:** 2020-02-19

**Authors:** Sutanu Bhattacharya, Debswapna Bhattacharya

**Affiliations:** 1 Department of Computer Science and Software Engineering, Auburn University, Auburn, AL 36849 USA; 2 Department of Biological Sciences, Auburn University, Auburn, AL 36849 USA

**Keywords:** Protein folding, Protein structure predictions

## Abstract

The development of improved threading algorithms for remote homology modeling is a critical step forward in template-based protein structure prediction. We have recently demonstrated the utility of contact information to boost protein threading by developing a new contact-assisted threading method. However, the nature and extent to which the quality of a predicted contact map impacts the performance of contact-assisted threading remains elusive. Here, we systematically analyze and explore this interdependence by employing our newly-developed contact-assisted threading method over a large-scale benchmark dataset using predicted contact maps from four complementary methods including direct coupling analysis (mfDCA), sparse inverse covariance estimation (PSICOV), classical neural network-based meta approach (MetaPSICOV), and state-of-the-art ultra-deep learning model (RaptorX). Experimental results demonstrate that contact-assisted threading using high-quality contacts having the Matthews Correlation Coefficient (MCC) ≥ 0.5 improves threading performance in nearly 30% cases, while low-quality contacts with MCC <0.35 degrades the performance for 50% cases. This holds true even in CASP13 dataset, where threading using high-quality contacts (MCC ≥ 0.5) significantly improves the performance of 22 instances out of 29. Collectively, our study uncovers the mutual association between the quality of predicted contacts and its possible utility in boosting threading performance for improving low-homology protein modeling.

## Introduction

The problem of predicting the accurate three-dimensional (3D) structure of a protein from its amino acid sequence, known as the protein structure prediction problem, remains open^1^. Template based modeling, one of the most accurate approaches for structure prediction, utilizes homologous structural templates deposited in Protein Data Bank (PDB)^2^ to address this problem. In the absence of close homology, remote homology detection technique known as threading is one of the most reliable and robust strategies for predicting the 3D structure of a query protein^3–5^. Various threading methods have been developed during the last decade^3,5–18^ with noteworthy successes. Alongside, steady growth in sequence and structure^2^ databases in conjunction with the rapid development of statistical and computational methods for co-evolutionary sequence analysis coupled with deep learning have resulted in substantial progress in sequence based prediction of residue-residue contact information^19–34^. Consequently, residue-residue contact or distance has become valuable new information to explore in boosting the accuracy of protein threading. State-of-the-art threading methods such as EigenTHREADER^35^, map_align^36^ and DeepThreader^37^ recently revisit the idea of recognizing remote homology by incorporating inter-residue contact map or distance map information into threading. Specifically, Jones and coworkers developed EigenTHREADER that integrates contact information predicted by MetaPSICOV^20^ with a standard threading technique. Baker and coworkers developed map_align, which integrates co-evolutionary contacts (Gremlin^38^) with a threading-based method and subsequently use double dynamic programming^39^. Xu and coworkers developed DeepThreader, which integrates sequential features with inter-residue distance information.

Very recently, we have developed a new contact-assisted threading method by successfully integrating accurate residue-residue contact information for improved protein threading^40^. Specifically, we have integrated residue-residue contact maps predicted by RaptorX^26,41–43^, one of the most accurate contact prediction methods, with structural and sequential information such as profiles, secondary structure, solvent accessibility, torsion angles (psi and phi), and hydrophobicity for contact-assisted threading. Experimental results have shown that the inclusion of contact information attains statistically significantly better performance compared to contact-free threading method when everything else remains the same, demonstrating that the inclusion of contact information in protein threading is a promising avenue for improving the performance of threading method. Furthermore, in a head-to-head performance comparison utilizing the same RaptorX-derived contact maps to guarantee a fair comparison, our method has successfully outperformed state-of-the-art contact-assisted threading methods EigenTHREADER and map_align, indicating our method as one of the best contact-assisted protein threading protocols. However, it is not clear how the quality of a predicted contact map affects contact-assisted threading. Nor it is clear whether contact-assisted threading with low-quality contact maps is as advantageous over pure threading as contact-assisted threading with high-quality contact maps such as those predicted from RaptorX. Finally, in the presence of competing contact maps of comparable qualities predicted by state-of-the-art contact predictors, is there any advantage of using one over the other in terms of improved threading performance? While assessing the efficacy of contact maps for low-homology protein modeling requires a head-to-head comparison between contact-assisted threading and contact-free pure threading, neither EigenTHREADER nor map_align can perform threading in a contact-free mode. Our method, on the other hand, can be seamlessly customized to perform contact-assisted or contact-free threading modes, enabling the evaluation of the utility of contact maps for remote homology modeling.

To evaluate the significance of contact maps in low-homology protein modeling, here we systematically investigate the impact of the quality of predicted contacts on the accuracy of contact-assisted threading by employing our newly developed contact-assisted threading method over several datasets. First, we analyze predicted contact maps from RaptorX and three other complementary methods having a wide range of qualities of their predicted contacts based on different contact map evaluation criteria to objectively evaluate how to select the most informative contact map. Then, we integrate the predicted contact maps from these contact predictors one by one into our contact-assisted threading method to examine the impact of each predicted contact map on the threading performance and compare them with a baseline threading algorithm that does not utilize contact information as well as RaptorX-assisted threading. Finally, we compare the performance of our contact-assisted threading by incorporating comparable-quality contact maps predicted by the top two officially ranked contact predictors from the recently concluded 13th Critical Assessment of protein Structure Prediction (CASP13) experiment to further study the impact of the quality of contacts in threading performance. Collectively, our study unravels the mutual association that exists between the quality of a contact map and the performance of contact-assisted threading.

## Methods

### Scoring a query-template alignment

Our newly-developed contact-assisted threading method, described in^40^, is an iterative query – template alignment approach where query-template alignments are performed by a Needleman-Wunsch global alignment algorithm^44^. The threading scoring function consists of close and distant sequence profiles, secondary structure, solvent accessibility, structure profile, torsion angles, and hydrophobicity match based on which a normalized alignment score or Z_*score*_ is calculated for ranking the templates.

Residue-residue contact map, which is a binary, square, symmetric matrix, is a two-dimensional representation of protein’s 3D structure. A contact indicates that the spatial distance between a pair of residues is less than a given distance threshold, typically set at 8 Å, between the *C*_*α*_ or *C*_*β*_ atoms of the residue pairs. Contact Map Overlap (CMO) finds the similarity between two contact maps, where the higher CMO score indicates that a higher similarity between the two comparing contact maps. Al-Eigen^45^, one of the state-of-the-art CMO methods, computes an overlap between two input contact maps and gives a score between [0,1] with higher score indicating better agreement of contact maps. We integrate CMO score returned from Al-Eigen into our threading method for selecting the best-fit template by formulating the final score as discussed in^40^. After identifying the best-fit template, the query-template alignment is used to copy the coordinate of the aligned residues from the template to build the final 3D model of the query protein. Please refer^40^ for further details about our method and its scoring function.

### Template libraries, benchmark data, and predicted contact maps

We use a representative non-redundant library of templates containing 70,670 templates, collected from: https://zhanglab.ccmb.med.umich.edu/library/^46^.

Our first benchmark dataset is the PSICOV150 dataset^19^, which contains 150 single chain, single domain proteins. In order to test the impact of different types of contact maps in the performance of contact-assisted method, we choose predicted contact maps from four complementary methods having a wide range of qualities of predicted contacts including (i) mean field direct coupling analysis (mfDCA)^22,23^, (ii) sparse inverse covariance estimation method (PSICOV)^19^, (iii) classical neural network-based meta approach (MetaPSICOV)^20^, and (iv) state-of-the-art ultra-deep learning model (RaptorX)^26,41–43^. Here, we give a brief introduction of each contact predictor. mfDCA, an advanced formulation of direct coupling analysis (DCA), is a statistical inference framework used to infer direct co-evolutionary couplings between pair of residues in multiple sequence alignments. Another Evolutionary Coupling Analysis (ECA) technique, PSICOV, uses sparse inverse covariance estimation for contact prediction. Although ECA methods are useful for predicting long-range contacts in the presence of a large number of sequence homologs, their accuracy is substantially poor if the number of sequence homologs is low^47^. In recent years, machine learning or deep learning-based methods boost the accuracy of contacts. One such contact predictor, MetaPSICOV, a meta predictor, which uses a two-stage neural network by combining outputs of several ECA classifiers. It was ranked as one of the best contact predictors in CASP11 and CASP 12^48^. Another contact predictor powered by deep learning, RaptorX, incorporates the entire protein ‘image’ as a context for prediction by utilizing a Residual Convolutional Neural Network, or ResNet. It was ranked as one of the best contact predictors in CASP12 and CASP13^28^.

We use the FreeContact package^22^ to obtain contact-maps predicted by mfDCA. Since the contact likelihood scores of mfDCA predicted contact maps are not normalized in the range [0,1], we normalize contact likelihood scores by dividing each score by the maximum likelihood score of a given predicted contact map. PSICOV and MetaPSICOV contacts are obtained directly from the MetaPSICOV benchmark dataset^20^. RaptorX contacts are collected by submitting jobs to the RaptorX online server (http://raptorx.uchicago.edu/ContactMap/^26,41–43^). Residue pairs with contact likelihood scores <0.5 are excluded to reduce noise in all predicted contact maps. To make a fair performance comparison, we use the same template library for all competing methods by excluding templates with sequence identity >30% to the query protein to remove close homologs. It should be noted that, unlike other contact predictors, RaptorX fails to predict contacts for two targets namely: 1tqhA and 1hdoA. We, therefore, consider 148 targets for the current benchmarking.

Next, we benchmark on CASP13 dataset officially released in December 2018. We consider 20 full-length targets in a total of 32 domains for which CASP organizers released experimental structures so far. We consider the top two officially ranked contact predictors in CASP13^49^ to test the impact of using comparable-quality contact maps in the performance of our contact-assisted method. In CASP13, the contact prediction category is heavily dominated by the latest breakthroughs in deep learning technologies. For example, G498 (ranked 1) or RaptorX-Contact, developed by Xu and coworkers, has attained the top performance since CASP12. It predicts residue-residue contacts using an ultra-deep learning model. It is worth mentioning that we also use RaptorX predicted contacts for our previous study^40^ as well as for benchmarking on PSICOV150 dataset for this current work. The second-ranked contact predictor, G032 or TripletRes, developed by Zhang and coworkers^34^, is implemented by a deep residual fully convolutional neural network with evolutionary coupling features from deep multiple sequence alignment.

For CASP13 benchmarking, the template library is curated before CASP13 started on May 1, 2018, which contains 69,041 template structures. For a fair comparison, we use the same template library for all competing methods. We have downloaded the predicted contact maps from the official website of CASP and subsequently exclude residue pairs with contact probability <0.5 from all predicted contact maps to reduce noise. It is also worth mentioning all residue pairs of a predicted contact map with contact likelihood of at least 0.5 with minimum sequence separation of 6 residues are considered for all experiments^40^.

### Evaluation criteria of contact maps, and the resulting contact-assisted 3D structures

We use the following evaluation measures to evaluate predicted contact maps: precision, coverage, mean false positive error, spread, and Matthews correlation coefficient (MCC)^28,50,51^. Precision is the percentage of correctly predicted contacts, ${Precision}=\frac{TP}{TP+FP}$, where TP represents true positives or correctly predicted contacts, and FP represents false positives or incorrectly predicted contacts. Coverage is the percentage of correctly predicted contacts with respect to the number of true contacts in the native contact map (N_c_), $Coverage=\frac{TP}{{N}_{c}}$. Mean false positive error is the mean of absolute deviation of all incorrectly predicted contacts and is calculated by: $Mean\,FP\,Error=\frac{1}{FP}\sum ({d}_{ij}-d)$, where d represents the distance threshold (usually 8 Å) and d_ij_ represents the true distance of an incorrectly predicted pair of contacts. Spread is calculated by: $Spread=\frac{1}{{N}_{c}}{\sum }_{i=1}^{{N}_{c}}\min \{dist({T}_{i}-P)\}$, where N_c_ represents the number of true contacts, T_i_ is a true contact, and $\min \{dist({T}_{i}-P)\}$ is the minimum Euclidean distance between true pair of contacts and predicted contact pairs. Matthews correlation coefficient (MCC) is calculated by: $MCC=\frac{TP\ast TN-FP\ast FN}{\sqrt{(TP+FP)(TP+FN)(TN+FP)(TN+FN)}\,}$, where TP, TN, FP, and FN represent true positive, true negative, false positive, and false negative respectively.

TM-score^52^ is used to evaluate the quality of the predicted 3D structure of query proteins with respect to the native (experimentally determined) structures. The value of TM-score lies in the range (0,1], where a higher score indicates better similarity. A TM-score >0.5 suggests a highly similar structure to the native fold^53^.

## Results and Discussions

### Robust assessment of qualities of predicted contact maps

To objectively evaluate the most informative contact map, we compare the performance of each predicted contact map from different perspectives using various contact evaluation measures^51^ over PSICOV150 dataset after excluding two targets (1tqhA and 1hdoA) for which RaptorX fails to predict contact maps. As shown in Table 1, mfDCA attains the highest precision of 75.22% compared to 72.83% of PSICOV, 72.08% of RaptorX, and 71.61% of MetaPSICOV. From the standpoint of precision, mfDCA seems to be the best contact predictors. However, all the other contact evaluation measures indicate that RaptorX attains the best performance. For example, RaptorX attains an MCC of 0.68 compared to 0.47 of MetaPSICOV, 0.24 of PSICOV, and 0.14 of mfDCA. RaptorX is also shown to reach the best score according to coverage (66.88%), mean floating point error (0.67), and spread (1.78), whereas, MetaPSICOV, PSICOV, and mfDCA achieve coverage of 34.2%, 8.78% and 3.2%, mean FP error of 0.73, 1.08 and 1.03, and spread of 5.63, 8.32 and 20.05 respectively. The results reveal that relying purely on one contact evaluation measure such as precision may not always be sufficient since evaluation measures focus on various aspects of the quality of predicted contacts that can sometimes be mutually contradictory. Furthermore, the fact that we only consider residue pairs with contact probability of at least 0.5 to remove noise, may have resulted in a very few numbers of contact pairs for mfDCA thereby artificially raising the precision. In contrast, MCC considers true and false positives and negatives, and therefore is a more balanced evaluation measure for predicted contacts.

**Table 1:** Evaluation of predicted contact maps^a^ on PSICOV150 dataset^b^, sorted by non-increasing order of the value of MCC (best performance and best performer are listed in bold).

Contact Source	Precision	Coverage	Mean FP Error	Spread	MCC
**RaptorX**	72.08	**66.88**	**0.67**	**1.78**	**0.68**
MetaPSICOV	71.61	34.20	0.73	5.63	0.47
PSICOV	72.83	8.78	1.08	8.32	0.24
mfDCA	75.22	3.20	1.03	20.05	0.14

^a^Excluding residue pairs with contact probability <0.5.

^b^Excluding two targets (1tqhA and 1hdoA) for which RaptorX could not predict contact maps.

**Figure 1:**
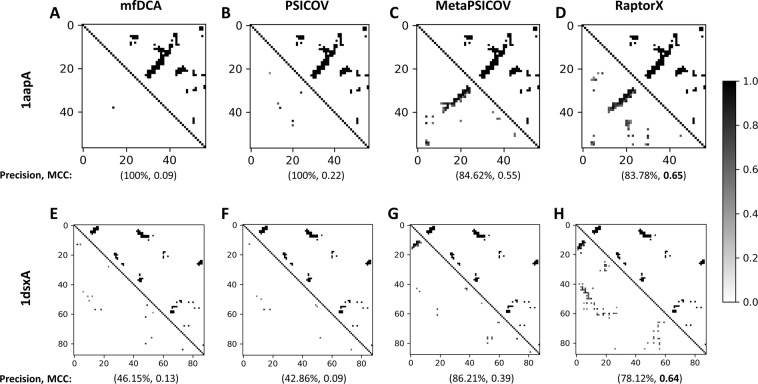
Representative examples of contact maps predicted by four complementary methods for targets 1aapA and 1dsxA. The upper triangles represent true (native) contacts of the target and the lower triangles represent predicted contacts with contact probability of at least 0.5. Numbers inside parenthesis indicate precision (%), and MCC respectively. For target 1aapA, (**A**) native contacts versus mfDCA contacts with a precision of 100% and an MCC of 0.09, (**B**) native contacts versus PSICOV contacts with a precision of 100% and an MCC of 0.22, (**C**) native contacts versus MetaPSICOV contacts with a precision of 84.62% and an MCC of 0.55, (**D**) native contacts versus RaptorX contacts with a precision of 83.78% and an MCC of 0.65. For target 1dsxA, (**E**) native contacts versus mfDCA contacts with a precision of 46.15% and an MCC of 0.13, (**F**) native contacts versus PSICOV contacts with a precision of 42.86% and an MCC of 0.09, (**G**) native contacts versus MetaPSICOV contacts with a precision of 86.21% and an MCC of 0.39, (**H**) native contacts versus RaptorX contacts with a precision of 78.12% and an MCC of 0.64.

As a representative example, we present two case studies on targets 1aapA (56 residues) and 1dsxA (87 residues) to illustrate mutual comparisons between precision and MCC, and to substantiate how MCC is more balanced evaluation measure for predicted contacts. In Fig. 1, the upper triangles represent native contact map and the lower triangles represent predicted contact map by different contact predictors after applying contact likelihood score cutoff of at least 0.5. Fig. 1(A,B,C,D) represent native contacts of the target 1aapA versus contacts predicted by mfDCA, PSICOV, MetaPSICOV, and RaptorX respectively. Based on precision, mfDCA and PSIOCOV appear to be the best contact predictor for the target 1aapA with a precision of 100%, as opposed to 84.62% of MetaPSICOV and 83.78% of RaptorX. However, mfDCA and PSICOV achieve high precision by predicting only a very few contact pairs correctly, but with very low coverage. Precision of MetPSICOV and RaptorX, on the other hand, are comparatively lower due to the presence of few false positive contacts, but with substantially higher coverage compared to mfDCA or PSICOV. MCC successfully addresses this issue with RaptorX achieving the best performance having an MCC of 0.65 compared to 0.55 of MetaPSICOV, 0.22 of PSICOV, and 0.09 of mfDCA. These results illustrate the fact that MCC is more balanced evaluation measure and therefore better suited for predicted contact maps that are often noisy. Fig. 1(E,F,G,H) present a similar case study for target 1dsxA (87 residues). Once again, RaptorX predicted contact map achieves the best performance in terms of MCC with a value of 0.64 compared to 0.39 of MetaPSICOV, 0.13 of mfDCA, and 0.09 of PSICOV; whereas MetaPSICOV contacts achieves the best performance in terms of precision (86.21%) compared to 78.12% of RaptorX, 46.15% of mfDCA, and 42.86% of PSICOV. Although this time precision offers better balance, still it overly emphasizes prediction of true positive contacts. Overall, these examples demonstrate that MCC is more robust and consistent for noisy contact maps compared to other contact evaluation measures. We, therefore, choose MCC as the main evaluation measure of the quality of predicted contact maps in this study.

### Performance evaluation of contact-assisted threading with contact maps of diverse qualities

To investigate the impact of the quality of contact maps on the performance of contact-assisted threading, we benchmark our method using contact maps of diverse qualities over PSICOV150 dataset. As shown in Table 2, our contact-assisted threading method powered by the high-quality contacts from RaptorX (referred to as rr_RaptorX_-assisted threading) and moderate-quality contacts from MetaPSICOV (referred to as rr_MetaPSICOV_-assisted threading) outperform contact-free pure threading method (referred to as pure threading) serving as a control in terms of the accuracy of the top ranked predicted models. Considering TM-score of top ranked models, RaptorX-assisted threading method delivers the best performance by achieving a mean TM-score of 0.66, which is 0.03 TM-score points more than that of baseline threading method, whereas the mean TM-score improvement reaches to 0.01 for MetaPSICOV-assisted threading method compared to baseline threading method. Moreover, 80.4% and 77.7% of the time RaptorX-assisted threading method and MetaPSICOV-assisted threading method predict the correct fold (TM-score >0.5), respectively, as opposed to 75.7% of baseline threading method. We also perform T-Test to examine whether the performance boost attained by contact-assisted threading work using high-quality RaptorX contacts and moderate-quality MetaPSICOV contacts over baseline threading method are statistically significantly better. Compared to baseline threading method, RaptorX-assisted threading method is statistically significantly better at 95% confidence level with a *p*-value of 0.0001. However, MetaPSICOV-assisted threading method improves the threading performance compared to baseline threading method, but the improvement is not statistically significant at 95% confidence level with a *p*-value of 0.07 (Supplementary Table S5). Overall, the results demonstrate that the threading method using high-quality contact maps leads to better threading performance in terms of TM-score of top ranked models and percentage of time finding the correct overall folds.

**Table 2:** Performance comparison on PSICOV150 targets^a^ based on top ranked models, sorted by non-decreasing order of performance (best performance and best performer are listed in bold) with shaded row representing the performance of pure threading method.

Methods	Average TM-score (*p*-value*)	%time TM-score >0.5^b^
rr_mfDCA_-assisted threading^c^	0.58 (1.5e-11)	69.6
rr_PSICOV_-assisted threading^d^	0.59 (1.3e-09)	71.6
pure threading^e^	0.63 (0.0001)	75.7
rr_MetaPSICOV_-assisted threading^f^	0.64 (0.0007)	77.7
**rr**_**RaptorX**_**-assisted threading**^g^	**0.66**	**80.4**

^a^Excluding two targets (1tqhA and 1hdoA) for which RaptorX could not predict contact maps.

^b^Percentage of time the respective method predicts the correct fold (TM-score > 0.5).

^c^Contact-assisted threading method using mfDCA contacts.

^d^Contact-assisted threading method using PSICOV contacts.

^e^Pure threading method (without contacts).

^f^Contact-assisted threading method using MetaPSICOV contacts.

^g^Contact-assisted threading method using RaptorX contacts.

*One sample T-Test’s *p*-value of the TM-score difference compared to rr_RaptorX_-assisted threading.

Table 2 also shows that low-quality contacts such as mfDCA and PSICOV degrade the contact-assisted threading (referred to as rr_mfDCA_-assisted threading and rr_PSICOV_-assisted threading respectively) performance with respect to pure threading method by 0.04 and 0.05 TM-score, respectively, in terms of the accuracy of top ranked predicted models. In finding correct overall folds, the performance of mfDCA-assisted threading method and PSICOV-assisted threading method drop by around 4% and 6%, respectively, compared to baseline threading method. The deterioration of performance of contact-assisted threading method using low-quality contact maps mfDCA and PSICOV are also statistically significant with *p*-values of 5.8e-08 and 2.9e-05, respectively with respect to baseline threading method (Supplementary Table S5). Moreover, Table 2 also shows that RaptorX-assisted threading attains statistically significantly better performance compared to the other three contact-assisted threading method, mfDCA-assisted threading, PSICOV-assisted threading, and MetaPSICOV-assisted threading, with *p*-values of 1.5e-11, 1.3e-09, and 0.0007 respectively. Results presented in Table 2, therefore, demonstrate that low-quality contacts degrade the threading performance compared to baseline threading method as opposed to high-quality contacts, which boost the threading performance.

**Figure 2:**
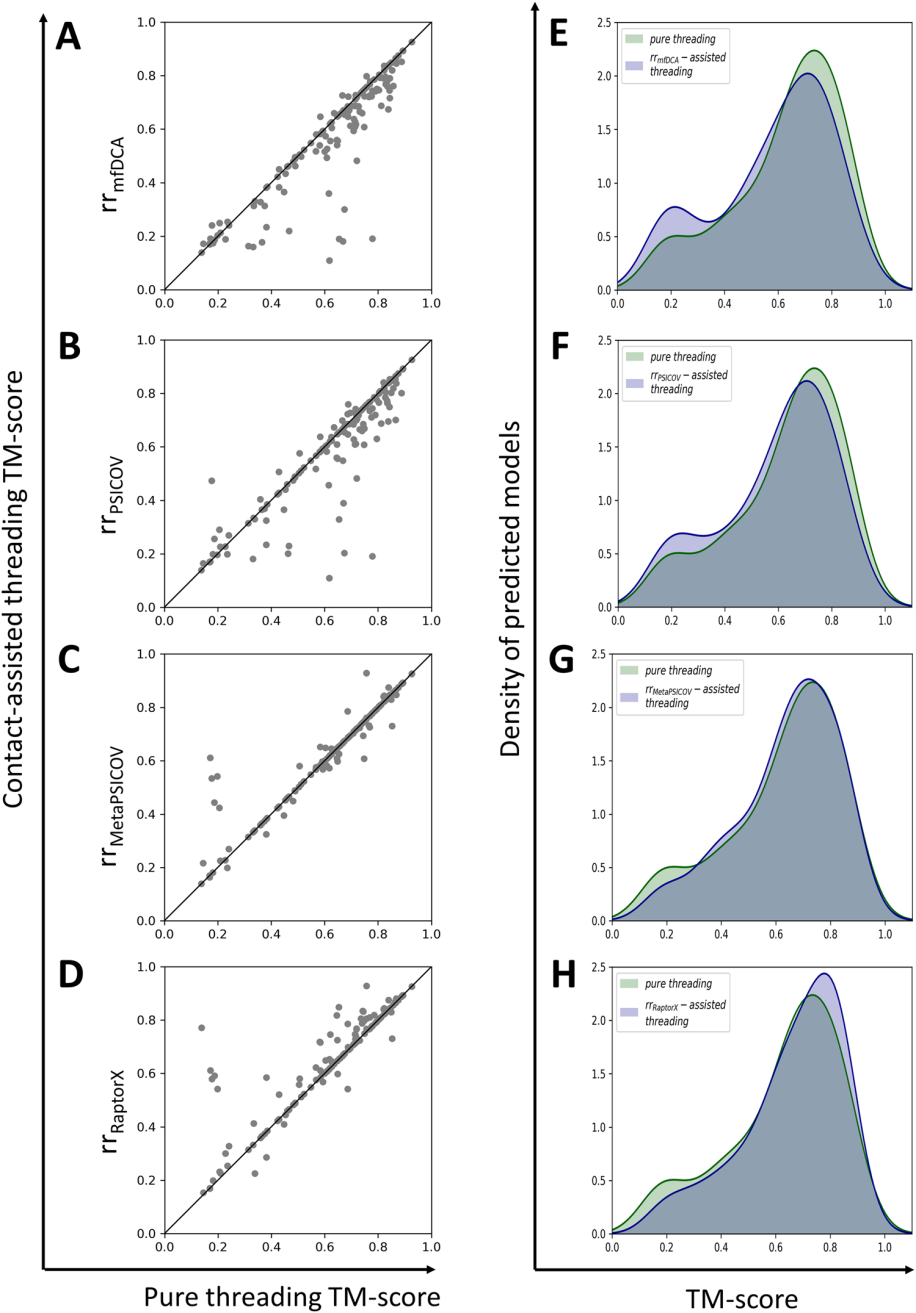
A head-to-head comparison of different contact-assisted threading methods and baseline contact-free pure threading method on PSICOV150 dataset. (**A**) mfDCA-assisted threading method (referred to as rr_mfDCA_) versus baseline threading method (referred to as Pure threading), (**B**) PSICOV-assisted threading method (referred to as rr_PSICOV_) versus baseline threading method, (**C**) MetaPSICOV-assisted threading method (referred to as rr_MetaPSICOV_) versus baseline threading method, (**D**) RaptorX-assisted threading method (referred to as rr_RaptorX_) versus baseline threading method. Each point in each scatter plot represents joint TM-score of top ranked model predicted by baseline pure threading method and contact-assisted threading method. (**E**) TM-score distribution of top ranked models predicted by pure threading method versus mfDCA-assisted threading method (referred to as rr_mfDCA_-assisted threading), (**F**) TM-score distribution of top ranked models predicted by pure threading method versus PSICOV-assisted threading method (referred to as rr_PSICOV_-assisted threading), (**G**) TM-score distribution of top ranked models predicted by pure threading method versus MetaPSICOV-assisted threading method (referred to as rr_MetaPSICOV_-assisted threading), (**H**) TM-score distribution of top ranked models predicted by pure threading based method versus RaptorX-assisted threading method (referred to as rr_RaptorX_-assisted threading). Templates with sequence similarity >30% to the query sequence are excluded.

Fig. 2 shows a head-to-head comparison of different contact-assisted threading methods with baseline contact-free threading method in terms of accuracy (TM-score) of top ranked models built from the first-ranked template. Each point in each scatter plot represents joint TM-score of top ranked model predicted by pure threading and contact-assisted threading method. In Fig. 2(A,B), majority of points are below diagonal lines, which clearly indicates that low-quality contacts (mfDCA and PSICOV) substantially degrade the threading performance compared to baseline threading method. In contrast, we observe a slight performance improvement using moderate-quality MetaPSICOV contacts (Fig. 2(C)), where MetaPSICOV-assisted threading method improves threading performance for 22 targets (out of 148) compared to pure threading method. Moreover, Fig. 2(D) shows a noticeable boost in threading performance using the high-quality RaptorX contacts, where 35.8% points (or 53 targets) are above the diagonal, indicating RaptorX-assisted threading method improves the TM-score of the top ranked model for 53 targets (out of 148) compared to baseline threading method. Furthermore, we examine the TM-score distribution of the top ranked model predicted by contact-assisted threading methods and baseline threading method in Fig. 2(E,F,G,H). Specifically, in Fig. 2(E,F), the highest peak of baseline threading method is larger as well as skewed towards the higher accuracy (right) side compared to mfDCA-assisted threading method and PSICOV-assisted threading method, respectively. These figures indicate that the threading method using each low-quality contact map predicts more models with low TM-score than baseline threading method, resulting bimodality due to the second highest peak of the density of predicted models in the TM-score range [0,0.4], which deteriorates the overall threading performance. In contrast, in Fig. 2(G,H), we see an opposite trend when we plot TM-score distribution of our threading approaches – one using contacts (MetaPSICOV, and RaptorX respectively) of higher qualities while the other does not use contact information. Fig. 2(G) shows a slight performance improvement by MetaPSICOV-assisted threading method compared to baseline threading method in that in TM-score range [0,0.3], MetaPSICOV-assisted threading method predicts fewer models as opposed to higher TM-score range, indicating using moderate contacts such as MetaPSICOV helps to improve the TM-score of a few targets compared to purely threading based method. In Fig. 2(H), we see a significant performance boost by incorporating the high-quality RaptorX contacts in threading method. The highest peak of RaptorX-assisted threading method is larger as well as skewed towards the higher TM-score (right) side compared to baseline threading method. In TM-score range [0.5,1.0], RaptorX-assisted threading method predicts more models as opposed to low TM-score range [0,0.5), indicating incorporating the high-quality RaptorX contacts helps to find the overall correct folds (TM-score >0.5) for a number of targets where purely threading based method fails. In summary, these results demonstrate that incorporating high-quality contacts in threading significantly boosts the threading performance in contrast with low-quality contacts, which degrades the performance.

**Figure 3:**
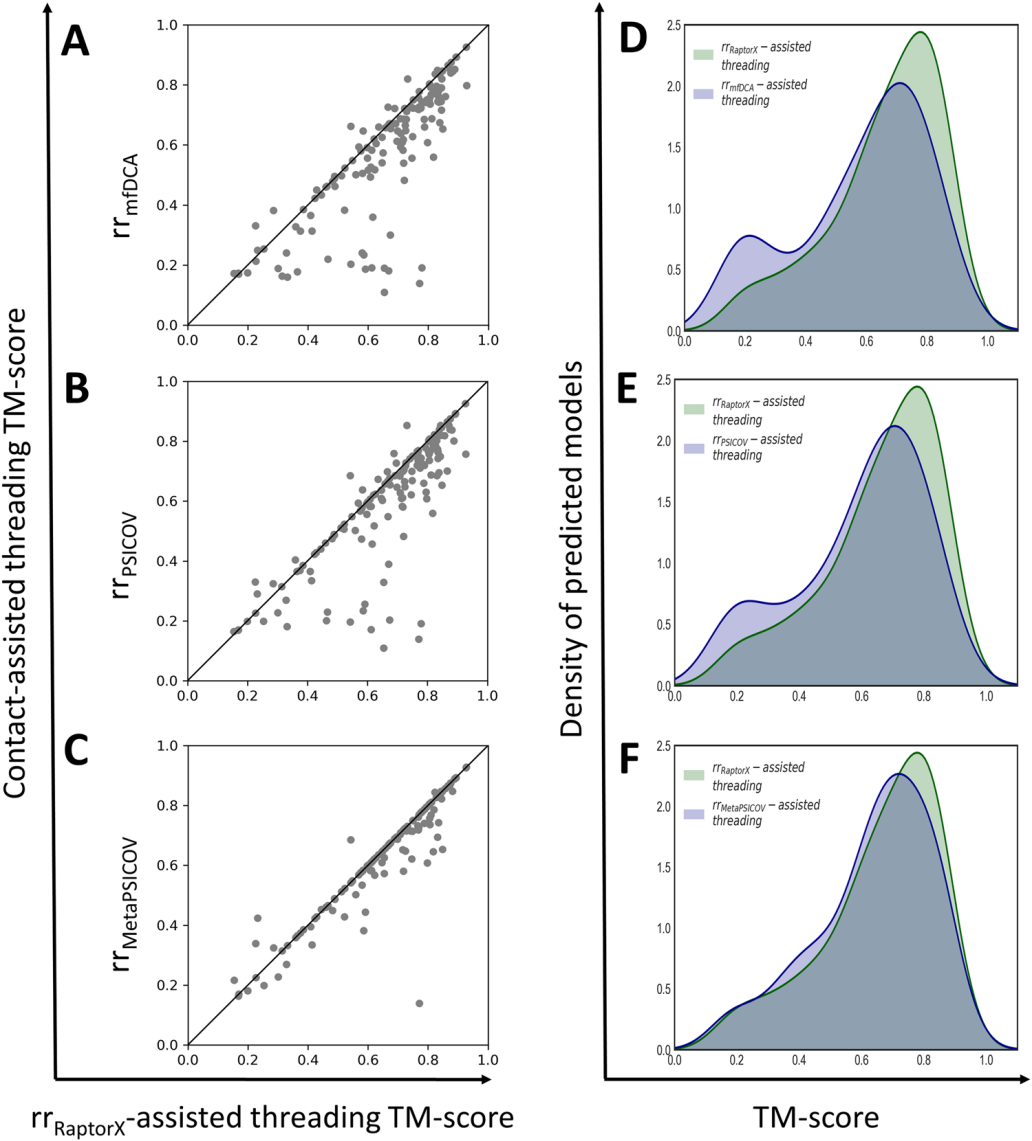
A head-to-head comparison of different contact-assisted threading methods and baseline RaptorX-assisted threading method on PSICOV150 dataset. (**A**) mfDCA-assisted threading method (referred to as rr_mfDCA_) versus baseline RaptorX-assisted threading method (referred to as rr_RAPTORX_-assisted threading), (**B**) PSICOV-assisted threading method (referred to as rr_PSICOV_) versus baseline RaptorX-assisted threading method, (**C**) MetaPSICOV-assisted threading method (referred to as rr_MetaPSICOV_) versus baseline RaptorX-assisted threading method. Each point in each scatter plot represents joint TM-score of top ranked model predicted by baseline RaptorX-assisted threading and one of the other three contact-assisted threading methods respectively. (**D**) TM-score distribution of top ranked models predicted by RaptorX-assisted threading method versus mfDCA-assisted threading method (referred to as rr_mfDCA_-assisted threading), (**F**) TM-score distribution of top ranked models predicted by RaptorX-assisted threading method versus PSICOV-assisted threading method (referred to as rr_PSICOV_-assisted threading), (**G**) TM-score distribution of top ranked models predicted by RaptorX-assisted threading method versus MetaPSICOV-assisted threading method (referred to as rr_MetaPSICOV_-assisted threading). Templates with sequence similarity >30% to the query sequence are excluded.

Since RaptorX-assisted threading method delivers the best threading performance we compare the performance of other three contact-assisted threading methods one by one against RaptorX-assisted threading acting as control. In Fig. 3, each point in each scatter plot represents TM-score of top ranked model predicted by RaptorX-assisted threading vs. one of the other three contact-assisted threading methods. Fig. 3(A) shows a head-to-head comparison of mfDCA-assisted threading (referred to as rr_mfDCA_) and RaptorX-assisted threading (referred to as rr_RaptorX_-assisted threading method) in terms of TM-score of top ranked model, where majority of the points (>70%) are below the diagonal line, RaptorX-assisted threading clearly outperforms mfDCA-assisted threading by a large margin. We see almost a similar trend in Fig. 3(B) when we compare PSICOV-assisted threading (referred to as rr_PSICOV_) with RaptorX-assisted threading method. Around 67% points are below the diagonal line, which demonstrates the superior performance of RaptorX-assisted threading over PSICOV-assisted threading. In Fig. 3(C), we compare our contact-assisted threading approaches – one using moderate-quality MetaPSICOV contacts (referred to as rr_MetaPSICOV_) while other using the high-quality RaptorX contacts. Around 28% more points are below the diagonal line, which illustrates the positive influence of higher-quality contact maps (RaptorX) for improved threading performance. In Fig. 3(D,E), compared to both mfDCA- and PSICOV-assisted threading methods (referred to as rr_mfDCA_-assisted threading and rr_PSICOV_-assisted threading respectively), the highest peak of RaptorX-assisted threading (referred to as rr_RaptorX_-assisted threading) is larger as well as skewed towards the higher TM-score side, indicating RaptorX-assisted threading finds more correct folds compared to others. Similarly, Fig. 3(F) illustrates the TM-score distribution of MetaPSICOV-assisted threading (referred to as rr_MetaPSICOV_-assisted threading) and RaptorX-assisted threading, where the highest peak of RaptorX-assisted threading is still larger than MetaPSICOV-assisted threading as well as skewed towards the higher accuracy (right) side, illustrating the performance boost attained by contact-assisted threading method using the high-quality contacts (RaptorX) over moderate-quality contacts (MetaPSICOV). Overall, high-quality contacts predicted from RaptorX leads to statistically significantly better threading performance compared to that attained from inferior-quality contacts predicted from other methods.

**Figure 4:**
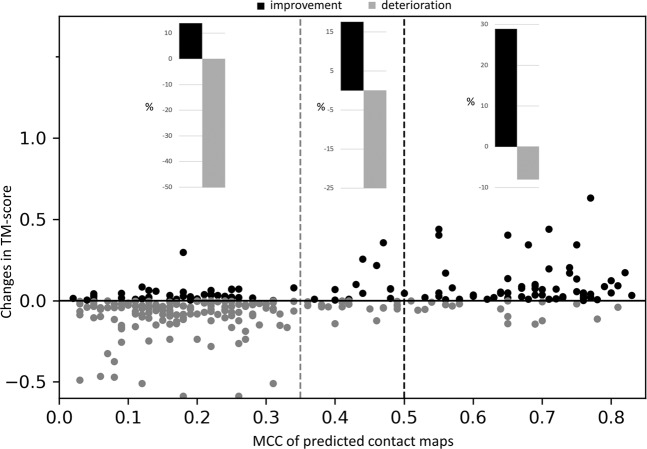
The relationship between changes in TM-score of contact-assisted threading methods compared to pure threading method, and the MCC (Matthews correlation coefficient) of predicted contact maps, tested on PSICOV150. The dataset includes all four contact-assisted threading methods over 148 targets resulting in a total of 592 instances. Each point in the scatter plot represents MCC of a predicted contact map and change in TM-score of a top ranked model predicted by various contact-assisted threading methods compared to pure threading. The dark points indicate improvement in TM-score (positive change in TM-score), whereas the grey points indicate performance deterioration (negative change in TM-score) compared to pure threading. The data points are separated based on the quality (measured by MCC by considering residue pairs with contact probability of at least 0.5) of contacts: (i) 211 pairs with high quality contacts (MCC ≥ 0.5), (ii) 301 pairs with low-quality (MCC < 0.35) contacts, and (iii) the twilight zone comprises of 80 pairs with moderate-quality contacts (0.35 ≤ MCC < 0.5). Each bar plot represents the percentage of TM-score improvement and deterioration compared to pure threading. Templates with sequence similarity >30% to the query protein are excluded.

Fig. 4 shows how the quality of contact maps (measured by MCC for residue pairs having contact probability of at least 0.5) affects contact-assisted threading performance as quantified by the changes in TM-score of top ranked models of contact-assisted threading methods compared to pure threading method considering all four contact-assisted threading methods over 148 targets resulting in a total of 592 instances. Each point in the scatter plot represents MCC of a predicted contact map and change in TM-score of a top ranked model predicted by the respective contact-assisted threading method compared to pure threading method respectively. The data points have been separated based on the quality (MCC) of contacts: (i) 211 pairs with high quality contacts (MCC ≥ 0.5), (ii) 301 pairs with low-quality (MCC < 0.35) contacts, and (iii) the twilight zone comprises of 80 pairs with moderate-quality contacts (0.35 ≤ MCC < 0.5). The bar plot on the upper right corner of Fig. 4 shows that contact-assisted threading performance is significantly improved for around 29% of the cases (out of 211), which is more than three times of the number of cases the performance is degraded, demonstrating that high-quality contacts (MCC ≥ 0.5) boost threading performance. In contrast, the bar plot on the upper left corner of Fig. 4 shows that low-quality contacts degrade contact-assisted threading performance for almost half of the points (out of 301) as opposed to only around 14% of the cases where the performance is improved, illustrating the adverse effect of low-quality contacts (MCC < 0.35) on contact-assisted threading performance. The bar plot on the upper middle section of Fig. 4 represents a twilight zone with moderate-quality contacts where there is no significant difference in the number of cases contact-assisted threading performance is improved (around 18%) or degraded (25%) out of 80 pairs. Furthermore, in Supplementary Fig. S1, targets are grouped into three bins based on their sequence length to investigate how the quality of contacts affects the changes in TM-score of contact-assisted threading compared to the baseline pure threading for different length bins. In Supplementary Fig. S1, there are 34 targets of length <100 residues resulting in a total of 136 instances (Fig. S1(A)), 47 targets of length [100,150] residues resulting in a total of 188 instances (Fig. S1(B)), and 67 targets of length >150 residues resulting in a total of 268 instances (Fig. S1(C)). For every length bin, we see a similar trend, contacts with MCC ≥ 0.5 lead to improved threading performance as opposed to contacts with MCC < 0.35, which degrade the threading performance. Specifically, in the presence of high-quality contacts (MCC ≥ 0.5), Fig. S1(A) shows the highest threading performance boost of ~37% for the small proteins followed by ~29% for proteins of length [100, 150] residues (Fig. S1(B)) and ~24% for proteins of length > 150 residues (Fig. S1(C)) compared to ~8% performance degradation in each distance bin. On the other hand, low-quality contacts (MCC < 0.35) degrade threading performance for ~50% of the cases, irrespective of protein length. Overall, the results show that contact maps with an MCC score of at least 0.5 lead to significantly better threading performance, whereas a score below 0.35 corresponds to a significant deterioration in threading performance.

**Figure 5:**
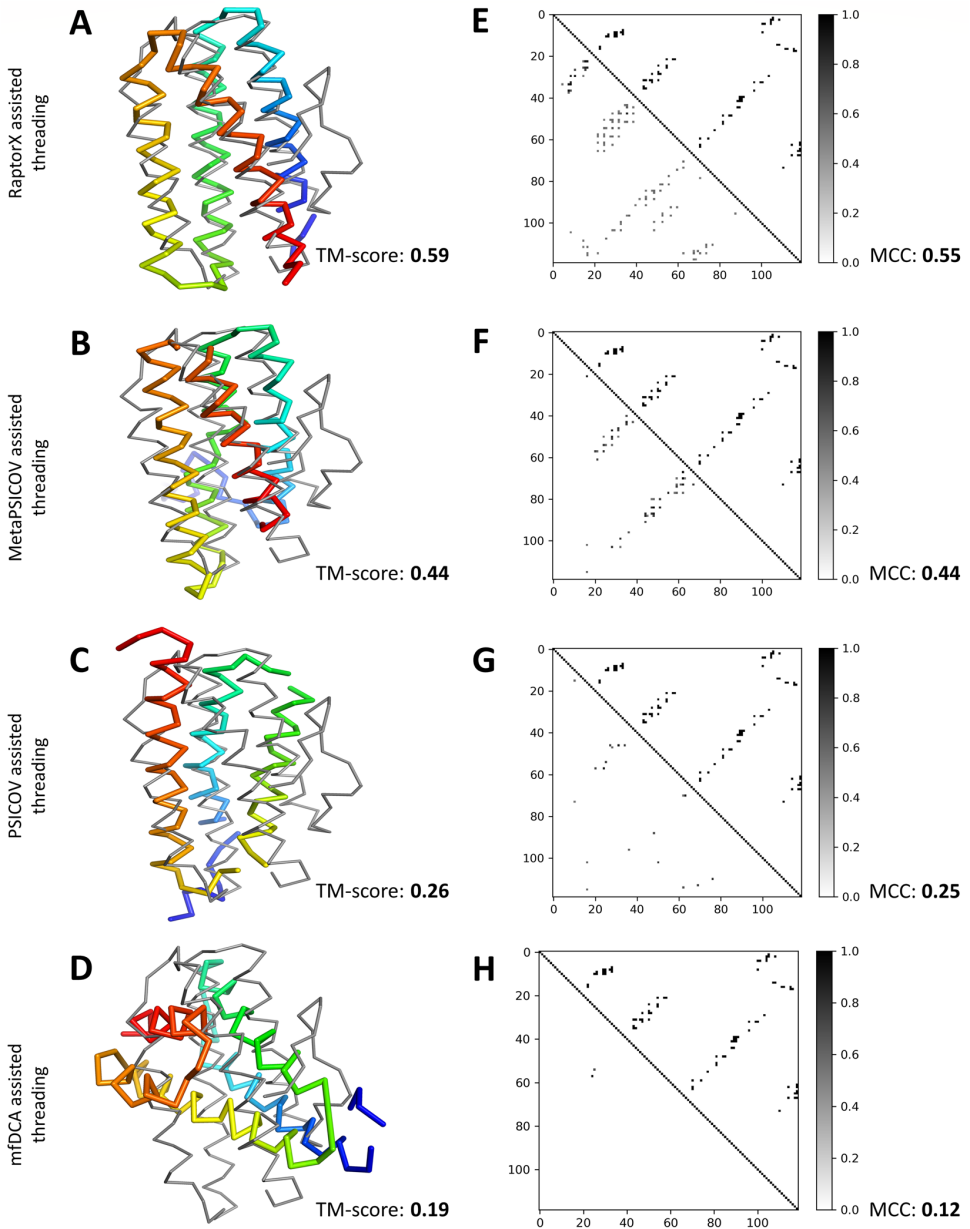
Representative example of contact-assisted threading with contact maps of diverse qualities on target 2mhrA. (**A**) Structural alignment between the top ranked model predicted by RaptorX-assisted threading (in thick rainbow) with a TM-score of 0.59 and the native structure of the target (in thin gray), (**B**) Structural alignment between top ranked model predicted by MetaPSICOV-assisted threading (in thick rainbow) with a TM-score of 0.44 and the native structure of the target (in thin gray), (**C**) Structural alignment between top ranked model predicted by PSICOV-assisted threading (in thick rainbow) with a TM-score of 0.26 and the native structure of the target (in thin gray), (**D**) Structural alignment between top ranked model predicted by mfDCA-assisted threading (in thick rainbow) with a TM-score of 0.19 and the native structure of the target (in thin gray). (**E**) Native contact map (upper triangle) versus predicted contact map by RaptorX (lower triangle) with an MCC of 0.55. (**F**) Native contact map (upper triangle) versus predicted contact map by MetaPSICOV (lower triangle) with an MCC of 0.44. (**G**) Native contact map (upper triangle) versus predicted contact map by PSICOV (lower triangle) with an MCC of 0.25. (**H**) Native contact map (upper triangle) versus predicted contact map by mfDCA (lower triangle) with an MCC of 0.12. For all predicted contact maps, pair of residues with contact probability <0.5 are excluded.

A representative example sheds some light on the impact of diverse quality of contacts on threading performance, as shown in Fig. 5 for target 2mhrA from the PSICOV150 dataset that is a Hemerythrin HHE cation binding domain^19^ of 118 residues. Fig. 5(A) shows RaptorX-assisted threading predicts the correct fold (top ranked model predicted with a TM-score >0.5) with a TM-score of 0.59 (and root-mean-square deviation or RMSD of 4.8 Å) by using RaptorX predicted contacts with an MCC of 0.55 (Fig. 5(E)). In contrast, Fig. 5(B,C,D) reveal the inability of the other three contact-assisted threading methods in finding the correct fold due to inferior-quality contacts. In particular, threading using moderate-quality MetaPSICOV contacts (MCC of 0.44, Fig. 5(F)) predicts the 3D structure of the target with a TM-score of 0.44 (and RMSD of 12.15 Å, Fig. 5(B)) while as shown in Fig. 5(C,D), TM-score (and RMSD) are 0.26 (and 11.85 Å) and 0.19 (and 13.48 Å) for method using PSICOV contacts (MCC of 0.25, Fig. 5(G)) and mfDCA contacts (MCC of 0.12, Fig. 5(H)) respectively.

### Performance evaluation of contact-assisted threading with contact maps from top CASP13 groups

To further study the effect of the quality of contacts in threading performance over challenging CASP13 targets, we employ contact-assisted threading using the top two officially ranked contact maps on CASP13 dataset, consisting of 20 full-length targets (and 32 domains) officially released so far with native structures, the same template library and the same nr sequence database, curated before CASP13 started on May 1, 2018, are used by all competing methods. For each target, we make the prediction for the full sequence without utilizing any domain information. After the prediction phase, threading performance at the domain level is evaluated using the domain definitions provided by the official CASP13 assessors.

Table 3 shows incorporating high-quality contacts statistically significantly outperforms the baseline pure threading method both for full-length targets and domain level targets. Over 20 full-length targets (and 32 domains), the mean TM-score of threading methods using TripletRes contacts (referred to as TripletRes-assisted threading) and RaptorX-Contact (referred to as RaptorX-Contact-assisted threading) are 0.457 (and 0.392) and 0.449 (and 0.387), respectively, as opposed to 0.403 (and 0.34) of the baseline pure threading method. Moreover, the performance improvement of TripletRes and RaptorX-Contact are also statistically significant with *p*-value of 0.001 (and 0.0002) and 0.006 (and 0.0008), respectively, for full-length (and domain level) targets. Additionally, Supplementary Fig. S2 shows how threading performance is affected by the quality of contacts over 20 full-length targets. The set contains 40 instances, out of which, there are 29 instances with high-quality contacts (MCC ≥ 0.5) as opposed to only one instance (TripletRes contact map for T1008) for which MCC < 0.35. The figure demonstrates how high-quality contacts with MCC ≥ 0.5 lead to significant threading performance boost (22 out of 29), illustrating contact maps with an MCC score of at least 0.5 lead to significantly better threading performance. A case study shown in Supplementary Fig. S3 for CASP13 target T0954 of length 350 residues demonstrates the impact of high-quality contacts on threading performance. The baseline pure threading method attains TM-score of 0.301 for the target, whereas contact-assisted threading using high-quality contact maps (MCC ≥ 0.5) from RaptorX-contact and TripletRes successfully predict the correct fold with TM-score ≥ 0.56, illustrating how high-quality contacts with MCC ≥0.5 boost threading performance.

**Table 3:** Performance evaluation on CASP13 dataset^a^ based on average TM-score of top ranked models.

Target type	TripletRes- assisted threading (*p*-value*)^b^	RaptorX-Contact- assisted threading (*p*-value*)^c^	pure threading^d^
Full-length	0.457 (0.001)	0.449 (0.006)	0.403
Domain level	0.392 (0.0002)	0.387 (0.0008)	0.340

^a^CASP officially released 20 full-length targets in a total of 32 domains on December 2018.

^b^Zhang and coworkers participated in CASP13 with TripletRes (group number G032).

^c^Xu and coworkers participated in CASP13 with RaptorX-Contact (group number G498).

^d^Pure threading method (without contacts).

*One sample T-Test’s *p*-value of the TM-score difference compared to pure threading.

## Conclusions

Protein threading represents one of the most successful approaches for modeling protein 3D structures from sequences, particularly when close homologous structural templates cannot be easily detected. Emerging methods in protein co-evolution coupled with deep learning have shown promise in sequence-based prediction of protein residue-residue contact maps, which are valuable source of information that can facilitate further progress in protein threading. Very recently, we have successfully incorporated contact maps to boost the accuracy of protein threading, demonstrating contact-assisted threading as a promising avenue for remote-homology protein modeling^40^. However, the nature of the interdependence between the quality of contact maps and contact-assisted threading performance remains elusive. Here, we present a large-scale analysis to study their mutual association by employing contact-assisted threading using contact maps of diverse qualities predicted from various contact predictors ranging from pure co-evolutionary methods (mfDCA and PSICOV) to hybrid approaches that combine sequence co-evolution and machine learning such as classical neural network (MetaPSICOV) and ultra-deep learning model (RaptorX). Experimental results demonstrate that contact-assisted threading method using high-quality RaptorX contacts and moderate-quality MetaPSICOV contacts outperform the baseline contact-free threading, whereas, low-quality contacts predicted from mfDCA and PSICOV deteriorate the threading performance compared to the baseline pure threading method. Contact-assisted threading with the best-quality contacts (RaptorX) delivers the best threading performance that is statistically significantly better compared to contact-free threading, demonstrating that accurate (MCC ≥ 0.5) residue-residue contact information is highly effective in boosting threading performance as opposed to low-quality (MCC < 0.35) contact information. This holds true even on the recently concluded CASP13 dataset, where contacts with MCC ≥ 0.5 lead to improved threading performance. Collectively, our study shows that contact-assisted threading is effective in the presence of high-quality (MCC ≥ 0.5) contact maps – indicating an evolving new direction for improved protein threading that is likely to mature further with future advancements in contact prediction methods.

## Supplementary information

Supplementary Information.

## Data Availability

All data generated or analyzed during this study are included in this article and its supplementary files. Moreover, PSICOV150 dataset is publicly available at http://bioinfadmin.cs.ucl.ac.uk/downloads/PSICOV/suppdata/, CASP13 dataset is publicly available at http://www.predictioncenter.org/casp13/index.cgi, and contact maps predicted by top ranked groups in CASP13 are publicly available at http://www.predictioncenter.org/download_area/CASP13/predictions/contacts/.
