# Supplementary material for: Evaluating the significance of contact maps in low-homology protein modeling using contact-assisted threading
Source: Sci Rep. 2020 Feb 19;10:2908. doi: 10.1038/s41598-020-59834-2 (PMC7031282; doi:10.1038/s41598-020-59834-2)
Supplement: Supplementary file 1 — Supplementary Information. [file 41598_2020_59834_MOESM1_ESM.pdf]

Evaluating the significance of contact maps in low-homology protein modeling using contact-assisted threading

Sutanu Bhattacharya[1], Debswapna Bhattacharya[1,2*]

[1]Department of Computer Science and Software Engineering, [2]Department of Biological Sciences, Auburn University, Auburn, AL 36849, USA.

*To whom correspondence should be addressed. Phone: (334) 844-6321. Fax: (334) 844-6329. E-mail: bhattacharyad@auburn.edu.

Supplementary Figure S1. The relationship between changes in TM-score of contact-assisted threading methods compared to the baseline pure threading method, and the MCC of predicted contact maps, tested on PSICOV150. The targets are grouped into three bins based on their sequence length. (A) 34 targets of sequence length < 100 residues are considered. This set includes 136 instances, considering all four contact-assisted threading methods. (B) 47 targets of sequence length [100,150] residues are considered, resulting in a total of 188 instances by considering all four methods. (C) 67 targets of sequence length >150 residues are considered, resulting in a total of 268 instances by considering all four contact-assisted threading methods. See Figure 4 in the main manuscript for more explanation.

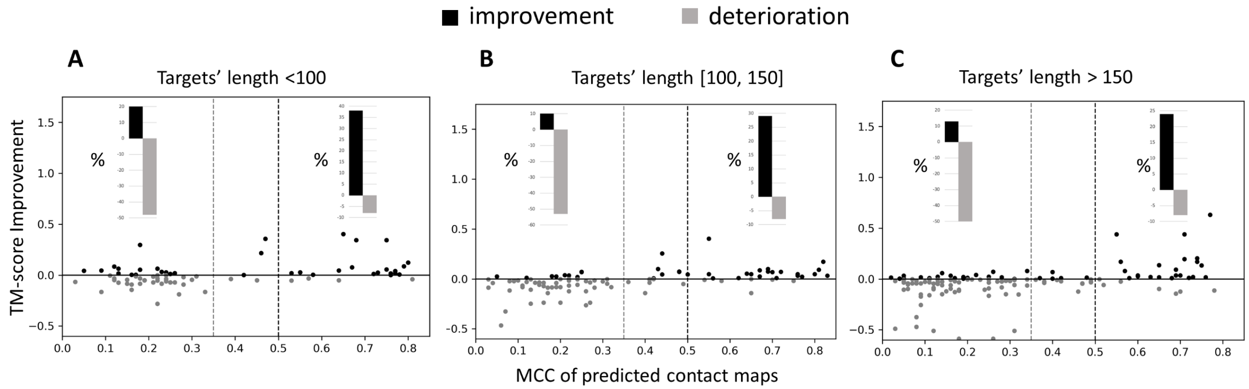

Supplementary Figure S2. The relationship between changes in TM-score of contact-assisted threading methods compared to the baseline pure threading method, and the MCC of predicted contact maps, tested on officially released 20 full-length targets of CASP13. This set includes a total of 40 instances, considering two contact-assisted threading methods. Out of these, there are 29 instances with high-quality contacts (MCC ≥ 0.5) as opposed to only one instance (TripletRes contact map for T1008) with an MCC < 0.35. An instance for which there is a change in TM-score (either positive or negative) compared to the baseline pure threading is only plotted. See Figure 4 in the main manuscript for more explanation.

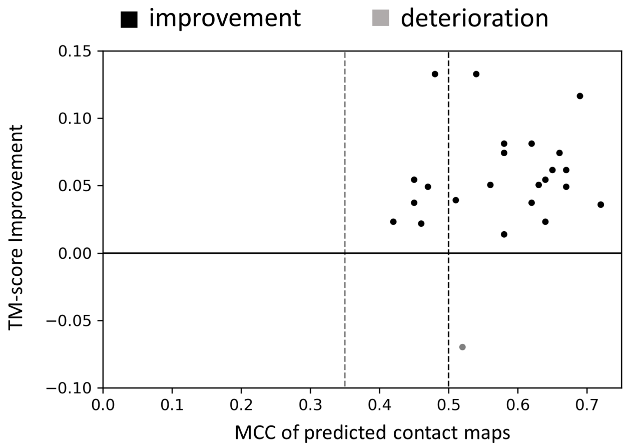

Supplementary Figure S3. A representative example of contact-assisted threading with top2 officially ranked contact predictors of CASP13 on target T0954. (A) Structural alignment between the top ranked model predicted by pure threading method (in thick red) with a TM-score of 0.301 and the native structure of the target (in thin gray), (B) Structural alignment between top ranked model predicted by threading methods using high-quality (MCC ≥ 0.5) contacts, TripletRes (in thick salmon red) and RaptorX-contact (in thick limon yellow), with TM-score of > 0.56 and the native structure of the target (in thin gray).

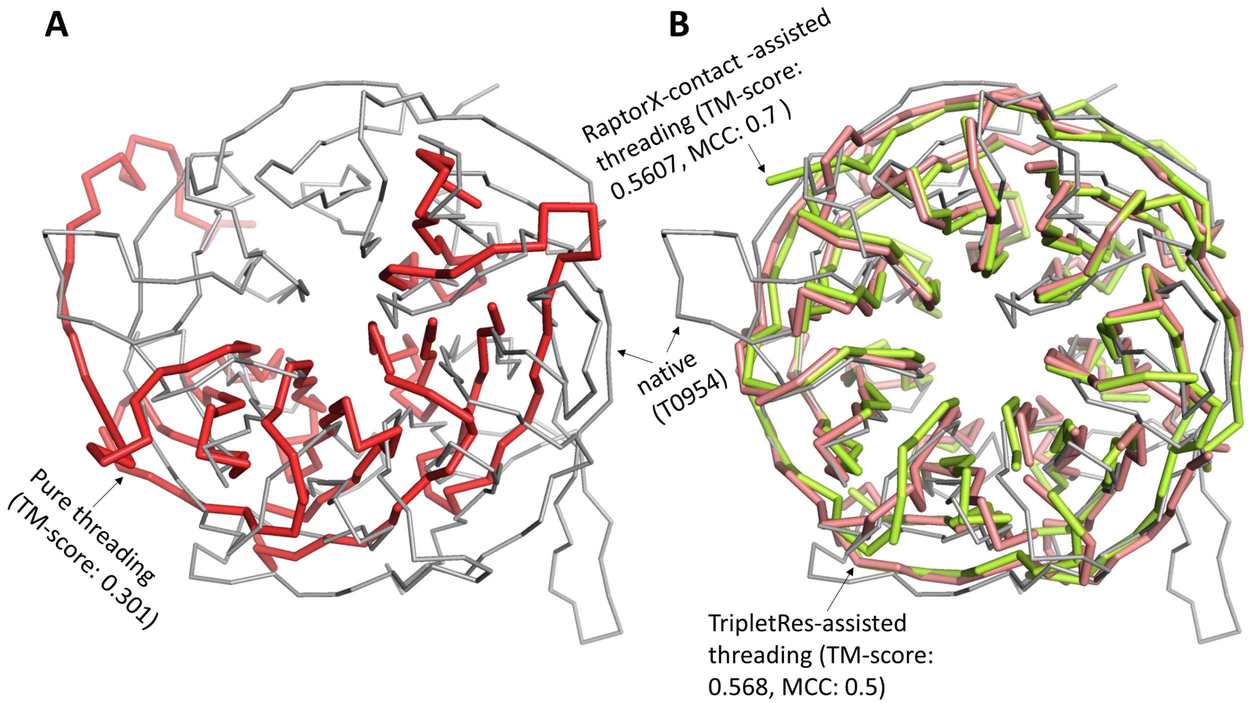

Supplementary Table S1. Evaluation of contact maps on PSICOV 148 targets*

Target	PSICOV_rr					mfDCA_rr					metaPSICOV_rr					RaptorX_rr				
	prec	cov	m_e	spread	mcc	prec	cov	m_e	spread	mcc	prec	cov	m_e	spread	mcc	prec	cov	m_e	spread	mcc
1bdoA	94.44	8.85	0.21	5.9	0.28	33.33	0.52	0.91	39.52	0.03	79.63	44.79	0.32	1.64	0.58	83.8	78.12	0.45	1.34	0.8
1aoeA	52.44	10.7	1.3	3.39	0.23	33.33	0.25	1.79	56.19	0.03	62.89	30.35	0.89	3.42	0.43	60.87	69.4	0.88	1.04	0.64
1jkxA	63.64	12.1	1.93	6.85	0.27	75	3.24	0.46	21.07	0.15	73.24	44.92	0.61	3.07	0.57	73.31	81.86	0.47	0.8	0.77
1i4jA	90	7.83	0.25	8.25	0.26	82.35	6.09	2.04	8.29	0.22	76.92	39.13	0.38	2.07	0.54	63.73	81.74	1.12	0.48	0.71
1wkcA	65.15	11.41	1.18	3.29	0.27	55.56	3.98	1.39	11.86	0.14	73.71	41.64	0.64	2.89	0.54	75.99	61.27	0.83	0.93	0.67
1d0qA	68.75	12.57	0.52	3.41	0.28	80	4.57	0.17	11.94	0.19	72.04	38.29	0.47	2.45	0.51	66.2	80.57	0.49	0.84	0.72
2phyA	76.92	7.75	0.5	18.46	0.24	70.59	4.65	0.96	18.8	0.18	65.69	25.97	0.81	6.33	0.4	65.7	43.8	0.75	4.34	0.52
1ny1A	76.64	15.33	0.42	11.29	0.34	75	0.56	0.29	38.91	0.06	71.24	39.81	0.63	7.78	0.53	65.31	65.79	0.78	4.73	0.65
1nb9A	75.51	12.46	0.89	8.67	0.3	100	0.67	0	29.75	0.08	67.16	46.13	1.12	3.85	0.55	65.71	76.77	1.09	2.07	0.7
1gzcA	70.27	3.59	1.92	9.4	0.15	40	0.28	3.51	46.26	0.03	64.96	24.55	1.42	7.07	0.39	79.96	59.86	0.59	1.74	0.69
1f6bA	69.77	8.31	1.04	6.08	0.24	55.56	1.39	1.56	19.26	0.08	65.49	25.76	1.16	7.08	0.4	68.77	51.25	0.99	2.17	0.58
1smxA	72.73	4.65	0.58	7.19	0.18	73.33	6.4	0.79	4.99	0.21	50.77	38.37	2.91	2.13	0.42	81.82	73.26	0.36	1.15	0.76
1atlA	52	2.87	2.18	10.73	0.12	50	0.44	0.51	34.77	0.05	53.73	7.95	0.91	14.76	0.2	79.4	58.72	0.34	1.44	0.68
1jo8A	100	10.24	0	3.69	0.31	100	3.15	0	8.91	0.17	78.57	69.29	0.35	0.61	0.71	82.91	76.38	0.15	0.8	0.78
1ag6A	88.24	6.33	0.09	11.18	0.23	100	1.69	0	24.87	0.13	66.38	32.49	0.85	4.99	0.44	67.47	70.89	0.58	1.08	0.67
1ctfA	88.89	6.35	0.06	7.05	0.23	100	2.38	0	12.61	0.15	82.14	36.51	0.25	1.84	0.53	79.41	85.71	0.24	0.49	0.81
1vp6A	87.93	18.48	0.3	6.8	0.4	90	3.26	1.18	18.31	0.17	78.87	55.43	0.3	2.91	0.65	51.33	63.04	3.31	1.4	0.55
1avsA	64.71	12.5	0.71	5.4	0.28	75	3.41	0.45	15.56	0.16	63.83	34.09	0.77	3.01	0.45	60.19	70.45	0.83	0.88	0.64
1ckeA	80.49	16.58	0.72	4.58	0.36	100	2.76	0	17	0.16	68.22	44.22	0.7	2.29	0.54	72.33	78.14	1.14	0.82	0.75
1i1jA	71.43	5.88	1.98	7.92	0.2	33.33	0.39	3.48	38.26	0.03	74.42	25.1	0.91	6.2	0.42	77.65	54.51	0.45	2.44	0.64
1i1nA	74.75	12.98	0.74	4.47	0.31	100	1.05	0	35.57	0.1	62.71	39.82	1.4	4.98	0.49	71.93	47.19	0.74	4.71	0.57
1fx2A	76	13.29	1.9	6.02	0.31	64.29	6.29	0.67	8.87	0.2	62.14	44.76	0.92	3.6	0.52	50.3	59.44	3.63	2.26	0.53
1c9oA	100	5.16	0	9.76	0.22	70	4.52	2.15	6.61	0.16	85.58	57.42	0.18	1.27	0.68	82.35	72.26	0.21	1.25	0.75
1ne2A	74.55	9.36	1.7	15.09	0.26	80	2.74	1.5	25.58	0.14	57.02	31.51	1.58	8.43	0.41	82.75	59.13	0.41	3.27	0.69
1mk0A	87.5	3.87	0.56	11	0.18	72.22	7.18	1.12	8.49	0.22	69.77	33.15	0.67	7.18	0.47	71.79	61.88	0.43	3.23	0.65
1cjwA	72.31	13.47	0.52	8.43	0.31	60	2.58	0.42	12.75	0.12	70.55	32.95	0.51	5.51	0.47	73.91	68.19	0.35	2.87	0.7
1kq6A	58.33	6.42	1.48	14.38	0.19	33.33	0.46	1.52	42.48	0.04	75.41	21.1	0.48	12.46	0.39	73.01	54.59	0.32	4.33	0.62
1c44A	50	1.21	4.58	14.91	0.07	100	0.4	0	47.87	0.06	73.02	18.62	0.55	7.32	0.36	71.9	70.45	0.66	1.02	0.7
1bkrA	71.43	3.16	0.19	12.69	0.15	65.52	12	0.92	5.94	0.27	76.32	18.35	0.31	3.93	0.37	70.3	73.42	0.4	0.69	0.71
1gmiA	93.75	8.93	0.12	10.98	0.28	33.33	0.3	4.56	42.96	0.03	87.41	37.2	0.13	2.96	0.56	80.58	74.11	0.23	1.06	0.76
1chdA	80.68	12.63	0.57	3.89	0.31	85.71	1.07	0.23	34.24	0.09	71.93	43.77	0.68	2.41	0.55	65.79	70.82	0.83	0.91	0.67
1abaA	60	6.43	0.77	5.62	0.19	85.71	4.29	0.03	8.37	0.19	73.4	49.29	0.44	3.32	0.59	73.03	79.29	0.33	0.41	0.75
1jyhA	75	5.68	0.14	8.52	0.2	83.33	4.73	0.09	8.68	0.19	73.26	43.22	0.97	6.5	0.55	69.21	74.45	0.73	0.69	0.71
2mhrA	72.22	8.84	1.73	7.91	0.25	100	1.36	0	39.9	0.12	78.72	25.17	0.56	11.46	0.44	64.81	47.62	1.73	1.59	0.55
3dqgA	80	9.73	1.2	4.89	0.27	87.5	2.13	0.01	28.66	0.13	80.65	37.99	0.29	3.48	0.54	67.76	75.38	0.89	1.28	0.7

1rybA	75.95	13.19	0.55	3.37	0.31	85.71	5.27	0.18	9.98	0.21	80.57	30.77	0.42	2.76	0.49	74.9	81.76	0.38	0.69	0.78
1roaA	80	1.61	0.02	15.41	0.11	64	6.45	2.71	7.66	0.19	70.33	25.81	0.6	3.76	0.41	73.43	80.24	0.55	0.59	0.76
1m8aA	100	3.16	0	14.26	0.17	60	9.47	0.6	3.2	0.22	74.19	48.42	0.37	2.18	0.58	79.31	72.63	0.18	0.68	0.74
1a6mA	75	4.5	0.85	10.36	0.18	33.33	1	2.52	32.44	0.05	87.23	20.5	0.15	6.37	0.42	68.28	77.5	0.49	0.46	0.72
1xkrA	50	1.62	4.23	14.27	0.09	16.67	1.85	7.77	10.95	0.05	69.84	10.19	0.72	26.11	0.26	59.02	72.69	2.22	0.53	0.65
2tpsA	68.37	12.76	0.96	5.42	0.29	80	2.29	0.32	15.39	0.13	72.16	45.71	0.59	3.28	0.57	73	72.95	0.46	2.33	0.72
1eazA	92.59	11.9	0.13	3.31	0.32	90	4.29	0.08	13.07	0.19	73.61	50.48	1.33	2.71	0.6	84.69	79.05	0.14	0.62	0.81
1jbkA	37.93	3.1	3.33	12.1	0.1	64.71	3.1	1.63	14.3	0.14	72.62	17.18	0.74	10.79	0.35	73.2	63.1	0.66	1.01	0.67
1ej0A	69.23	6.51	0.95	5.32	0.21	80	2.89	0.13	11.63	0.15	70.87	35.18	0.54	2.49	0.49	75.06	71.08	0.37	1.02	0.72
1jfxA	70.27	9.58	0.66	7.29	0.25	80	0.74	0.03	41.12	0.08	70.92	32.78	0.66	4.04	0.47	71.95	68.51	0.6	1.66	0.69
1jo0A	57.14	2.55	0.37	9.25	0.12	50	0.64	0.63	23.6	0.05	76.67	29.3	0.32	4.6	0.46	65.02	84.08	0.54	0.61	0.73
1gbsA	66.67	12.31	1.49	18.09	0.28	72	4.62	0.82	17.01	0.18	68.22	22.56	0.8	15.81	0.38	71.01	37.69	0.81	11.19	0.51
1dsxA	42.86	2.17	1.99	9.52	0.09	46.15	4.35	2.48	8.11	0.13	86.21	18.12	0.23	6.67	0.39	78.12	54.35	0.53	2.6	0.64
1pkoA	76.32	9.6	0.27	6.25	0.26	100	0.66	0	33.43	0.08	75.12	51.99	0.58	2.73	0.61	74.04	69.87	0.43	1.71	0.71
2cuaA	83.33	7.99	0.43	10.48	0.25	100	2.24	0	18.65	0.15	80.51	30.35	0.35	8.85	0.48	80	61.34	0.25	5.91	0.69
1qf9A	61.22	8.57	1.32	6.41	0.22	76.92	2.86	0.19	19.59	0.15	60.87	32	0.76	3.8	0.43	71.88	70.86	0.43	0.98	0.71
1k6kA	78.95	6.49	0.23	5.16	0.22	60	2.6	2.04	12.51	0.12	86.21	21.65	0.25	6.24	0.43	71.76	67.1	0.59	0.92	0.69
1g9oA	93.33	7.57	0.1	7.3	0.26	87.5	3.78	0.36	12.09	0.18	68.97	43.24	1.56	1.67	0.53	78.89	76.76	0.28	0.51	0.77
1i71A	75	3.37	0.47	8.53	0.15	100	2.81	0	10.69	0.16	77.08	20.79	0.45	3.83	0.38	67.02	35.39	1.25	3.08	0.47
1fcyA	41.94	3.44	2.88	21.18	0.12	4.26	1.59	8.39	29.65	0.02	77.5	8.2	0.55	25.28	0.25	80.95	44.97	0.22	7.47	0.6
1fl0A	67.27	8.79	0.98	13.7	0.24	90.48	4.51	0.21	17.67	0.2	64.06	29.22	1.1	11.8	0.42	75.78	63.18	0.36	2.5	0.68
1vmbA	79.17	9.55	0.14	3.51	0.27	100	1.51	0	15.19	0.12	81.25	38.69	0.55	2.03	0.55	75.11	87.44	0.22	0.25	0.8
1tifA	88.89	6.9	1.18	4.75	0.24	100	6.03	0	6.08	0.24	78.33	40.52	0.5	1.73	0.55	76.38	83.62	0.27	0.38	0.79
1im5A	69.49	9.36	1.93	6.87	0.25	100	4.11	0	19.65	0.2	73.27	33.79	0.95	5.02	0.49	74.76	71.69	0.36	0.85	0.72
1i5gA	57.14	8	0.7	10.19	0.21	75	2	0.48	18.05	0.12	62.5	33.33	0.55	7.5	0.44	53.33	50.67	1.3	3.8	0.5
1k7jA	69.86	9.66	0.68	5.86	0.25	75	0.57	1.27	41.87	0.06	74.5	28.22	0.61	4.47	0.45	66.18	77.46	0.76	0.7	0.71
1h98A	70	5.11	0.49	11.4	0.18	38.1	5.84	2.27	9.06	0.13	53.57	21.9	1.05	5.2	0.32	59.3	37.23	0.65	6.67	0.45
1pchA	90.48	10.27	0.04	4.48	0.3	76.92	10.8	0.48	3.81	0.28	79.09	47.03	0.63	2.29	0.59	73.47	77.84	0.32	0.82	0.74
1atzA	81.82	7.5	0.32	5.62	0.24	88.89	6.67	0.15	6.9	0.24	80	30	0.28	3.08	0.48	67.18	73.33	0.69	0.6	0.69
1whiA	88	6.71	0.17	7.88	0.24	92.31	3.66	0.02	7.96	0.18	78.46	31.1	0.58	4.14	0.48	79.56	66.46	0.3	1.33	0.71
1m4jA	33.33	0.77	4.55	15.71	0.05	47.37	3.47	3.22	10.34	0.12	63.72	27.8	0.71	6.53	0.41	68.08	68.34	0.45	0.87	0.67
1h2eA	80	18.38	0.4	3.8	0.38	92.31	5.25	0.02	19.49	0.22	73.99	44.2	0.46	2.14	0.56	73.25	75.27	0.35	0.95	0.74
1j3aA	79.17	7.57	0.57	11.99	0.24	85.71	4.78	0.23	15	0.2	73.56	25.5	0.85	3.54	0.42	62.86	52.59	3.11	2.36	0.56
1d4oA	52.08	6.19	2.91	7.12	0.17	71.43	1.24	0.23	28.72	0.09	71.19	10.4	1.08	11.39	0.27	67.86	61.14	1.72	1.69	0.63
1ej8A	56.52	3.51	2.17	11.1	0.13	100	0.81	0	30.09	0.09	56.99	28.65	1.57	3.46	0.39	70.34	50	1	3.63	0.58
1htwA	86.36	11.34	0.11	3.88	0.31	87.5	4.18	0.07	16.54	0.19	73.99	38.21	0.43	3.56	0.52	71.53	60	0.62	1.21	0.65
1o1zA	79.31	18.4	0.32	2.96	0.38	68.97	4	0.41	10.53	0.16	69.45	48.2	0.59	1.66	0.57	78.47	78	0.36	0.71	0.78
1vfyA	100	5.5	0	7.27	0.23	58.33	6.42	2.05	4.34	0.18	49.21	28.44	1.91	2.01	0.35	68.92	46.79	0.91	1.96	0.55
1cc8A	100	8.03	0	5.63	0.28	81.82	6.57	0.5	5.66	0.22	76.11	62.77	0.52	0.9	0.67	74.32	80.29	0.57	0.62	0.76

1t8kA	81.82	8.33	0.11	8.01	0.25	66.67	1.85	0.41	22.71	0.11	91.67	20.37	0.1	3.61	0.42	74.29	72.22	0.35	0.99	0.72
1bebA	41.67	1.31	3.9	14.22	0.07	34.04	4.19	4.3	8.69	0.11	68.54	31.94	0.77	7.04	0.46	67.04	63.35	0.95	1.66	0.64
1lm4A	76	12.39	0.54	7	0.3	80	0.87	0.2	31.77	0.08	55.74	22.17	1.36	5.97	0.34	62.35	55.43	1	2.93	0.58
1jvwA	86.15	16.05	0.18	4.15	0.37	90	2.58	0.06	17.8	0.15	71.13	57.88	0.67	2.37	0.63	76.06	71.92	0.29	1.67	0.73
1a3aA	90.74	15.08	0.2	4.52	0.36	100	3.08	0	15.44	0.17	77.93	34.77	0.39	3.63	0.51	67.41	74.46	0.76	0.9	0.7
1a70A	81.25	5.91	0.21	10.67	0.21	58.33	3.18	0.39	14.11	0.13	78.64	36.82	0.49	3.55	0.52	71.36	66.82	0.35	2.24	0.67
1kidA	71.43	5.76	1.54	8	0.2	66.67	0.46	0.11	39.25	0.05	84.62	17.74	0.62	9.18	0.38	69.81	75.12	0.63	1.83	0.72
1hxnA	51.52	3.31	3.79	32.69	0.13	19.33	10.1	6.4	13.64	0.12	74.4	24.32	0.61	15.86	0.42	74.73	54.09	0.57	2.93	0.63
1cxyA	90.91	7.63	0.08	7.04	0.26	100	3.82	0	9.59	0.19	79.25	32.06	0.39	3.06	0.49	85.05	69.47	0.25	1.54	0.76
1vjkA	88.24	7.81	0.6	7.55	0.25	100	1.56	0	13.05	0.12	89.23	30.21	0.17	4.08	0.51	80.34	74.48	0.36	0.75	0.76
1g2rA	80	7.55	0.56	5.36	0.24	100	1.89	0	14.95	0.13	78.05	20.13	0.42	4.28	0.39	76.22	78.62	0.35	0.61	0.76
1svyA	70	3.12	2.11	11.56	0.14	58.82	4.46	1.96	9.12	0.15	70.42	21.88	0.63	6.31	0.38	81.5	62.5	0.22	2.09	0.7
1dqgA	28.57	0.58	6.42	18.81	0.04	31.4	7.8	4.38	11.5	0.14	45.89	19.36	2.35	13.06	0.28	77.13	49.71	0.6	3.16	0.61
1guuA	85.71	13.33	1.02	6.09	0.33	100	2.22	0	13.27	0.15	86.96	44.44	0.35	1.34	0.61	76.19	71.11	0.26	0.61	0.72
1npsA	72.73	3.76	0.73	9.3	0.16	100	0.94	0	28.08	0.09	52.31	31.92	1.8	3.97	0.38	73.3	60.56	0.35	1.94	0.65
1ek0A	65.57	10.1	0.62	7.11	0.25	75	2.27	0.59	21.36	0.13	77.08	28.03	0.39	4.3	0.46	75.22	65.91	0.62	0.8	0.7
1dbxA	76.19	4.03	0.5	10.26	0.17	80	4.03	0.33	14.35	0.17	78.29	34.51	0.34	3.87	0.51	73.18	65.99	0.48	2.06	0.68
1josA	85	10.06	0.5	4.09	0.29	85.71	3.55	0.05	10.18	0.17	82.5	39.05	0.4	6.26	0.56	67.98	81.66	1.06	0.38	0.73
1jfuA	74.67	13.43	0.4	10.06	0.31	100	1.2	0	33.81	0.11	68.4	37.89	0.51	7.31	0.5	71.82	56.83	0.39	4.12	0.63
1c52A	42.86	2.54	1.78	18.96	0.1	40	2.54	2.95	19.98	0.09	59.09	16.53	0.98	6.01	0.3	80.18	37.71	0.28	3.39	0.54
1hfcA	70.83	5.01	0.97	9.15	0.18	100	0.29	0	68.1	0.05	67.74	12.39	0.79	8.66	0.28	76.6	53.1	0.43	2.27	0.63
1dixA	34.62	2.06	5.25	17.06	0.08	62.5	1.15	4.05	27.27	0.08	70.67	12.16	0.46	12.44	0.29	69.12	53.9	0.68	1.98	0.6
1mugA	66.67	3.97	0.86	8.73	0.16	48.39	4.25	1.5	6.9	0.14	86.15	15.86	0.14	8.53	0.36	66.25	60.06	0.68	1.47	0.62
1h0pA	76.92	11.86	0.43	4.92	0.29	82.61	3.75	0.13	12.63	0.17	63.8	41.11	0.81	3.6	0.5	78.04	66.01	0.29	2.2	0.71
1kw4A	83.33	6.25	0.78	5.27	0.22	100	5	0	9.15	0.22	66.67	27.5	0.5	2.49	0.41	76.83	78.75	0.22	0.32	0.77
1cznA	77.19	10.84	0.8	10.85	0.28	90	2.22	0.24	20.8	0.14	72.96	35.22	0.76	9.93	0.5	60.71	54.43	1.23	3.52	0.56
1i58A	56.47	13.33	1.95	7.52	0.27	30	0.83	2.94	29.8	0.05	73.49	33.89	0.54	3.08	0.49	65.56	49.17	3.56	1.66	0.56
1h4xA	60	7.73	1.43	9.63	0.21	71.43	5.15	0.47	11.11	0.19	65.57	41.24	0.46	3.96	0.51	59.49	72.68	0.85	0.56	0.64
1bsgA	74.31	12.37	0.62	4.49	0.3	76	2.9	0.67	21.15	0.15	76.7	32.67	0.4	5.59	0.49	73.81	61.53	0.59	1.73	0.67
1fvgA	73.49	13.26	0.38	6.89	0.31	85.71	2.61	0.07	16.35	0.15	62.59	38.91	0.99	6.23	0.48	66.44	63.7	0.64	2.11	0.64
3borA	72.22	11.9	1.18	4.39	0.29	87.5	1.6	1.21	40.02	0.12	77.46	30.66	0.43	3.56	0.48	75.87	64.76	0.33	1.05	0.69
1fvkA	65.38	10.33	1.45	14.14	0.26	100	0.3	0	58.33	0.05	68.21	31.31	0.7	8.74	0.46	63.86	62.31	1.39	1.25	0.62
1vhuA	71.21	9.77	0.73	4.3	0.26	75	1.25	0.57	17.54	0.09	68.69	42.41	0.74	2.08	0.53	64.72	73.6	0.65	0.6	0.68
1ku3A	80	14.04	0.33	4.42	0.33	77.78	12.3	0.37	4.51	0.3	67.44	50.88	0.51	1.04	0.57	75.38	85.96	0.43	0.21	0.8
1r26A	70.73	14.57	0.7	4.54	0.31	100	1.01	0	37.47	0.1	74.22	47.74	0.39	2.92	0.58	70.56	81.91	0.36	0.51	0.75
1fqtA	82.86	10.32	0.14	4.85	0.28	75	2.14	0.04	24.69	0.12	76.47	50.89	0.32	2.24	0.61	71.13	71.89	0.54	1.06	0.7
1d1qA	68.52	10.48	0.54	4.63	0.26	76.19	4.53	0.08	8.87	0.18	72.35	34.84	0.46	2.53	0.49	68.42	77.34	0.57	0.56	0.72
1p90A	60	4.71	2.67	7.82	0.16	75	2.35	0.32	15.54	0.13	62.5	29.41	1.02	4.81	0.41	71.64	75.29	0.45	1.39	0.72
1behA	69.23	3.77	0.95	15.11	0.16	66.67	1.26	0.76	19.88	0.09	76.72	18.45	0.59	12.61	0.37	63.16	52.62	1.09	3.31	0.57

2arcA	47.62	2.87	4.58	21.05	0.11	43.75	2.01	4.57	23.27	0.09	64.36	18.68	0.82	20.02	0.34	78.01	62.93	0.4	4.91	0.69
1jwqA	91.49	18.78	0.07	2.91	0.41	100	1.75	0	18.55	0.13	76.33	50	0.47	1.46	0.61	82.01	74.67	0.28	0.65	0.78
1tqgA	72.73	13.33	0.77	5.21	0.31	75	5	0.69	16.56	0.19	81.25	43.33	0.24	1.91	0.59	76.64	87.5	0.41	0.26	0.81
1kqrA	36.84	1.59	8.95	16.84	0.07	20	0.91	7.29	17.54	0.04	51.75	13.15	2.09	26.37	0.25	57.37	48.3	1.77	2.88	0.51
1iibA	86.67	6.5	0.15	6.61	0.23	69.57	8	1.05	4.6	0.23	66.67	41	0.63	2.76	0.51	68.88	83	0.49	0.34	0.74
1iwdA	76	13.87	0.66	4.06	0.32	100	0.91	0	32.38	0.09	66.22	35.77	1.07	2.73	0.48	77.8	61.86	0.4	1.28	0.69
1aapA	100	5.26	0	6.31	0.22	100	0.88	0	10.99	0.09	84.62	38.6	0.37	3.2	0.55	83.78	54.39	0.14	1.42	0.65
1qjpA	86.36	9.72	0.08	5.38	0.28	100	3.07	0	28.44	0.17	83.01	43.73	0.36	9.65	0.59	92.88	72.89	0.12	1.29	0.82
5ptpA	81.56	17.61	0.22	2.98	0.37	100	0.92	0	33.65	0.09	71.13	52.83	0.58	1.73	0.6	83.66	58.04	0.24	1.55	0.69
1rw7A	76.12	8.42	0.78	5.89	0.25	50	0.66	1.74	32.82	0.06	77.45	35.15	0.44	4.64	0.51	65.63	69.97	0.87	1.63	0.67
1xdzA	77.38	11.95	0.62	6.55	0.3	76.47	2.39	0.47	21.78	0.13	68.42	38.05	0.85	5.03	0.5	70.19	69.49	0.51	2.94	0.69
2vxnA	70.9	16.87	0.96	3.65	0.34	76.92	1.78	2.63	28.51	0.12	62.16	44.94	1.18	1.86	0.52	66.67	72.82	0.97	1.23	0.69
1dmgA	60.53	6.99	1.96	12.28	0.2	55.56	1.52	3.66	33.69	0.09	69.3	24.01	0.92	11.05	0.4	74.41	76.9	0.4	0.5	0.75
1brfA	100	3.3	0	10.62	0.17	100	7.69	0	8.91	0.27	55.56	43.96	1.26	1.34	0.46	82.54	57.14	0.25	4.61	0.67
1fk5A	66.67	1.43	0.96	17.58	0.09	40	4.29	5.27	5.42	0.12	40.35	16.43	2.69	5.96	0.24	70.08	63.57	0.54	0.85	0.66
2hs1A	60	5.71	0.9	3.72	0.17	18.67	6.67	6.54	3.68	0.09	100	0.48	0	34.92	0.07	78.75	60	0.32	1.32	0.67
1gz2A	67.39	9.31	0.35	4.19	0.24	100	1.2	0	26.86	0.11	72.96	34.83	0.69	2.87	0.49	69.84	66.07	0.53	1.19	0.67
1lo7A	75.93	14.29	1.64	5.29	0.32	71.43	8.71	2.27	7.23	0.24	79.08	54.01	0.72	2.44	0.64	77.08	90.24	0.24	0.44	0.83
1lpyA	58.33	11.81	1.01	6.24	0.26	80	1.69	0.03	25.41	0.11	42.86	21.52	1.96	7.39	0.29	58.53	63.71	0.82	1.09	0.6
1jl1A	76.92	11.56	0.19	4.38	0.29	100	2.02	0	16.85	0.14	75.33	32.66	0.3	2.82	0.49	76.99	72.54	0.42	0.87	0.74
1ihzA	73.81	10.03	0.74	4.98	0.26	75	1.94	0.74	15.62	0.12	84.8	34.3	0.31	4.55	0.53	76.68	70.23	0.36	1.86	0.72
1xffA	81.71	9.6	0.82	5.08	0.28	77.27	4.87	0.94	8.84	0.19	63.66	37.39	1.12	3.17	0.48	81.32	72.35	0.33	0.65	0.76
1jbeA	87.72	20.24	0.24	3.01	0.41	92.86	5.26	0.12	10.39	0.22	75.66	57.89	0.35	1.5	0.65	72.7	83	0.37	0.46	0.77
1nrvA	77.78	6.7	0.23	8.81	0.22	88.89	3.83	0.15	15.92	0.18	79.27	31.1	0.39	7.12	0.48	81.63	57.42	0.16	2.7	0.67
1fnaA	74.07	9.95	0.43	6.74	0.26	100	1.49	0	19.07	0.12	72.55	55.22	0.35	1.2	0.61	71.27	64.18	1.32	0.91	0.66
1k7cA	78.57	7.45	1.15	9.65	0.24	100	1.35	0	27.06	0.12	69.91	25.55	0.59	8.27	0.42	71.14	47.55	1.28	2.96	0.57
1hh8A	65.26	19.25	4.5	12.3	0.35	100	2.8	0	25.64	0.17	72.16	43.48	2.53	10.74	0.55	69.91	46.89	0.78	11.37	0.57
1ql0A	71.43	7.34	1.99	10.66	0.22	76.92	1.63	0.19	40.17	0.11	75.8	19.41	0.72	10.6	0.38	74.44	48.45	0.52	4.18	0.59
1tzvA	67.44	15.26	0.47	5.16	0.32	100	2.63	0	32.06	0.16	80.37	45.26	0.23	2.09	0.6	66.67	76.84	0.46	0.59	0.71
1gmxA	86.11	14.29	0.5	3.79	0.34	85.71	2.76	0.57	12.88	0.15	62.57	53.92	0.97	1.74	0.56	68.14	64.06	0.64	1.21	0.65
1ktgA	58.7	8.68	0.74	8.46	0.22	83.33	1.61	0.44	29.58	0.11	55.08	33.12	1.59	4.51	0.41	69.69	71.7	0.6	0.68	0.7
1dlwA	68.75	7.43	0.61	7.3	0.22	76.92	6.76	0.39	9.51	0.22	65.17	39.19	1.06	3.37	0.5	69.68	72.97	0.54	0.47	0.71
1wjxA	74.29	11.21	0.54	3.79	0.28	90.91	4.31	0.18	7.9	0.19	73.84	54.74	0.54	1.9	0.62	72.64	66.38	1.29	0.99	0.68
1w0hA	71.11	14.78	0.72	4.89	0.32	60	0.69	0.97	29.98	0.06	68.33	37.88	0.84	3.42	0.5	64.73	66.97	0.85	1.44	0.65
1rw1A	56.52	7.39	0.93	5.97	0.2	56.25	5.11	0.54	7.04	0.16	69.44	56.82	0.89	1.46	0.62	66.82	83.52	0.58	0.34	0.74
**Mean**	**72.83**	**8.78**	**1.08**	**8.32**	**0.24**	**75.22**	**3.20**	**1.03**	**20.05**	**0.14**	**71.61**	**34.19**	**0.73**	**5.63**	**0.47**	**72.08**	**66.88**	**0.67**	**1.78**	**0.68**

* excluding residue pairs with contact probability < 0.5 and excluding two targets (1tqhA and 1hdoA) for which RaptorX could not predict contact maps.

prec: precision, cov: coverage, m_e: mean_error

Supplementary Table S2. Performance evaluation of different methods on PSICOV 148 targets**

Target	Pure threading	Contact-assisted threading			
		PSICOV assisted	mfDCA assisted	metaPSICOV assisted	RaptorX assisted
1bdoA	0.6214	0.6214	0.5561	0.6214	0.7457
1aoeA	0.691	0.685	0.6716	0.691	0.691
1jkxA	0.1388	0.1388	0.1388	0.1388	0.7715
1i4jA	0.8116	0.688	0.688	0.8116	0.8116
1wkcA	0.8286	0.8042	0.7682	0.8286	0.8286
1d0qA	0.2279	0.2279	0.1885	0.2279	0.3006
2phyA	0.6	0.5973	0.5169	0.6	0.6
1ny1A	0.7755	0.7112	0.7228	0.7755	0.7755
1nb9A	0.7082	0.6845	0.5936	0.7082	0.7082
1gzcA	0.6685	0.5482	0.1806	0.6685	0.6685
1f6bA	0.7568	0.7568	0.7568	0.7616	0.7672
1smxA	0.5112	0.5112	0.4977	0.5119	0.5119
1atlA	0.8884	0.8018	0.8526	0.8884	0.8884
1jo8A	0.8646	0.8529	0.8529	0.8646	0.871
1ag6A	0.6476	0.6476	0.6476	0.6476	0.7251
1ctfA	0.4481	0.3655	0.3655	0.3951	0.409
1vp6A	0.7483	0.6082	0.6082	0.6082	0.7979
1avsA	0.649	0.5565	0.5565	0.5981	0.5981
1ckeA	0.3744	0.3691	0.313	0.3744	0.3744
1i1jA	0.5039	0.5026	0.5016	0.5026	0.5585
1i1nA	0.4896	0.4866	0.4622	0.4866	0.4896
1fx2A	0.6083	0.6075	0.5261	0.6083	0.6083
1c9oA	0.1979	0.1968	0.2028	0.5422	0.5422

1ne2A	0.6859	0.6859	0.662	0.6859	0.5423
1mk0A	0.1767	0.4732	0.2403	0.5342	0.5795
1cjwA	0.618	0.1098	0.1098	0.5733	0.6535
1kq6A	0.6875	0.6469	0.6469	0.6875	0.6982
1c44A	0.6543	0.3288	0.1894	0.6257	0.6543
1bkrA	0.7785	0.7434	0.7434	0.7785	0.7785
1gmiA	0.7138	0.7002	0.6277	0.7138	0.7475
1chdA	0.3142	0.3142	0.1634	0.3142	0.3142
1abaA	0.5671	0.5173	0.5173	0.5671	0.6221
1jyhA	0.1711	0.1711	0.1911	0.6117	0.6117
2mhrA	0.1866	0.2566	0.1866	0.4435	0.5914
3dqgA	0.4662	0.2293	0.2198	0.4662	0.4662
1rybA	0.4823	0.4746	0.4787	0.4492	0.4823
1roaA	0.7402	0.66	0.66	0.7402	0.7876
1m8aA	0.8438	0.8482	0.79	0.8482	0.8438
1a6mA	0.8681	0.8374	0.8454	0.8472	0.8813
1xkrA	0.1814	0.1989	0.1744	0.1814	0.1989
2tpsA	0.7942	0.7786	0.7469	0.7942	0.7942
1eazA	0.7428	0.7428	0.7428	0.7428	0.8347
1jbkA	0.3851	0.3851	0.3851	0.3851	0.3851
1ej0A	0.8654	0.8654	0.8654	0.8654	0.8654
1jfxA	0.7684	0.7349	0.7258	0.7258	0.7684
1jo0A	0.2064	0.2901	0.2499	0.4242	0.2321
1gbsA	0.6079	0.5827	0.4937	0.5827	0.6079
1dsxA	0.7194	0.7194	0.7194	0.7194	0.7658
1pkoA	0.6752	0.6547	0.6547	0.6752	0.6752
2cuaA	0.5056	0.5766	0.5056	0.581	0.581
1qf9A	0.653	0.653	0.653	0.653	0.8486
1k6kA	0.6858	0.7225	0.6858	0.7859	0.7859

1g9oA	0.8211	0.8409	0.79	0.8409	0.8409
1i71A	0.46	0.46	0.46	0.46	0.46
1fcyA	0.8226	0.8226	0.8362	0.8444	0.8226
1fl0A	0.5677	0.5677	0.581	0.5761	0.5761
1vmbA	0.7571	0.7571	0.7571	0.7571	0.8056
1tifA	0.2412	0.2698	0.2403	0.2698	0.328
1im5A	0.8045	0.8002	0.8008	0.8045	0.8045
1i5gA	0.6028	0.6028	0.5743	0.6491	0.6491
1k7jA	0.8657	0.7014	0.8657	0.8657	0.8657
1h98A	0.5834	0.638	0.6465	0.5834	0.5834
1pchA	0.667	0.667	0.667	0.667	0.667
1atzA	0.8338	0.7731	0.7794	0.8338	0.8338
1whiA	0.1453	0.1646	0.172	0.2162	0.1539
1m4jA	0.7156	0.7412	0.7269	0.7156	0.7412
1h2eA	0.757	0.757	0.7981	0.9278	0.9278
1j3aA	0.5911	0.5911	0.5911	0.597	0.597
1d4oA	0.5227	0.5227	0.5227	0.5227	0.5227
1ej8A	0.6263	0.624	0.6054	0.6263	0.6263
1htwA	0.3815	0.324	0.3815	0.324	0.2857
1o1zA	0.8567	0.8189	0.7612	0.8567	0.8567
1vfyA	0.6956	0.7273	0.6123	0.6956	0.6956
1cc8A	0.7185	0.7001	0.6862	0.7185	0.7185
1t8kA	0.7151	0.728	0.712	0.7151	0.728
1bebA	0.827	0.7851	0.7955	0.827	0.827
1lm4A	0.7071	0.688	0.688	0.7031	0.7071
1jvwA	0.6258	0.6365	0.6258	0.6459	0.6459
1a3aA	0.7362	0.7362	0.7362	0.7362	0.8058
1a70A	0.8355	0.7639	0.7608	0.8355	0.8355
1kidA	0.4884	0.4884	0.4955	0.4884	0.4884

1hxnA	0.4228	0.4228	0.4228	0.4228	0.4228
1cxyA	0.8394	0.8394	0.8394	0.8394	0.8394
1vjkA	0.7896	0.7199	0.7412	0.7896	0.7896
1g2rA	0.2362	0.1983	0.2535	0.1983	0.2535
1svyA	0.803	0.7656	0.7456	0.803	0.803
1dqgA	0.6579	0.6579	0.6579	0.6579	0.6579
1guuA	0.7951	0.6306	0.7505	0.7951	0.7951
1npsA	0.8379	0.6953	0.6741	0.8379	0.8379
1ek0A	0.8532	0.8532	0.8201	0.7312	0.7312
1dbxA	0.8436	0.8537	0.7162	0.8436	0.8436
1josA	0.5481	0.5481	0.5481	0.5481	0.5481
1jfuA	0.6741	0.6767	0.6708	0.6741	0.6741
1c52A	0.4528	0.4399	0.4341	0.4528	0.4447
1hfcA	0.7134	0.6096	0.6096	0.7134	0.7134
1dixA	0.6741	0.2034	0.3007	0.6741	0.6741
1mugA	0.6658	0.6987	0.7259	0.6658	0.6658
1h0pA	0.7217	0.7047	0.7217	0.7217	0.7217
1kw4A	0.7685	0.7685	0.7685	0.7685	0.8101
1cznA	0.749	0.6688	0.7234	0.749	0.749
1i58A	0.5937	0.5937	0.5937	0.5688	0.5688
1h4xA	0.6355	0.6734	0.6598	0.6355	0.6355
1bsgA	0.8932	0.8932	0.8932	0.8932	0.8932
1fvgA	0.7502	0.7502	0.7502	0.7502	0.7502
3borA	0.9263	0.9263	0.9263	0.9263	0.9263
1fvkA	0.6884	0.7598	0.7219	0.6884	0.6884
1vhuA	0.8039	0.8039	0.8039	0.8039	0.8039
1ku3A	0.7658	0.7658	0.7211	0.7333	0.7658
1r26A	0.8493	0.8024	0.8493	0.8303	0.8303
1fqtA	0.8467	0.8467	0.8467	0.8467	0.8467

1d1qA	0.779	0.1914	0.1914	0.779	0.779
1p90A	0.6467	0.6096	0.5407	0.6096	0.6467
1behA	0.7267	0.7181	0.685	0.7267	0.7267
2arcA	0.6151	0.457	0.3595	0.6151	0.6151
1jwqA	0.3384	0.3296	0.3314	0.3384	0.2257
1tqgA	0.8754	0.8754	0.8754	0.8754	0.8754
1kqrA	0.1695	0.1686	0.1732	0.1631	0.1686
1iibA	0.6201	0.6201	0.6201	0.6201	0.6201
1iwdA	0.3321	0.1812	0.1602	0.3321	0.3321
1aapA	0.6706	0.39	0.6706	0.6706	0.6706
1qjpA	0.6454	0.5597	0.5597	0.6454	0.8176
5ptpA	0.8397	0.8397	0.8397	0.8748	0.8748
1rw7A	0.8423	0.75	0.7447	0.8423	0.8423
1xdzA	0.4288	0.4288	0.4504	0.4288	0.4288
2vxnA	0.4284	0.5067	0.3831	0.4284	0.5212
1dmgA	0.5832	0.5832	0.5832	0.652	0.7163
1brfA	0.365	0.365	0.1773	0.365	0.365
1fk5A	0.3591	0.4037	0.3276	0.3591	0.3591
2hs1A	0.4886	0.4886	0.4886	0.4886	0.4886
1gz2A	0.7445	0.6859	0.7445	0.6943	0.8321
1lo7A	0.7856	0.7856	0.7856	0.7856	0.8195
1lpyA	0.583	0.583	0.583	0.583	0.612
1jl1A	0.3818	0.2344	0.2344	0.3818	0.5852
1ihzA	0.4628	0.2005	0.4628	0.4628	0.4628
1xffA	0.2095	0.2268	0.2131	0.2254	0.2268
1jbeA	0.8223	0.8206	0.8206	0.8219	0.8448
1nrvA	0.8928	0.8908	0.8928	0.8928	0.8928
1fnaA	0.8352	0.7646	0.8352	0.8352	0.8352
1k7cA	0.7166	0.6115	0.6184	0.7166	0.7166

1hh8A	0.3344	0.3344	0.3132	0.3344	0.4129
1ql0A	0.7694	0.7694	0.778	0.7694	0.7694
1tzvA	0.7094	0.6282	0.6379	0.7094	0.7094
1gmxA	0.719	0.719	0.719	0.719	0.768
1ktgA	0.7311	0.6659	0.6659	0.7238	0.7238
1dlwA	0.8075	0.8075	0.7924	0.8075	0.8075
1wjxA	0.1699	0.1699	0.1699	0.1699	0.1699
1w0hA	0.581	0.581	0.5399	0.581	0.7181
1rw1A	0.7199	0.4827	0.4827	0.7199	0.7199
**Mean**	**0.63**	**0.59**	**0.58**	**0.64**	**0.66**
**% TM-score > 0.5**	**75.7**	**71.6**	**69.6**	**77.7**	**80.4**

** excluding residue pairs with contact probability < 0.5 and excluding two targets (1tqhA and 1hdoA) for which RaptorX could not predict contact maps.

Supplementary Table S3. Performance evaluation of different methods on 20 CASP 13 full-length targets

Full-length Target	Pure threading	TripletRes(G032) assisted threading	RaptorX-Contact(G498) assisted threading
T0950	0.1494	0.1714	0.1635
T0951	0.9338	0.9697	0.9697
T0953s1	0.1703	0.2868	0.2097
T0953s2	0.095	0.2279	0.2279
T0954	0.301	0.568	0.5607
T0955	0.1605	0.2099	0.2099
T0957s1	0.2481	0.2481	0.1784
T0957s2	0.2536	0.2769	0.2769
T0958	0.3646	0.4019	0.4019
T0960	0.1061	0.1606	0.1606
T0963	0.1011	0.1517	0.1517
T0966	0.8081	0.8081	0.8081
T0968s1	0.2104	0.2847	0.2847
T0968s2	0.2041	0.2657	0.2657

T1003	0.8219	0.9033	0.9033
T1005	0.5692	0.5692	0.5692
T1008	0.389	0.389	0.389
T1009	0.8074	0.8074	0.8074
T1011	0.5615	0.5615	0.5615
T1016	0.802	0.8724	0.8724
**Mean**	**0.403**	**0.457**	**0.449**
**P-value**	-	0.001	0.006

Supplementary Table S4. Performance evaluation of different methods on 32 CASP13 domains

**Domain level Target**	**Pure threading**	**TripletRes(G032) assisted threading**	**RaptorX-Contact(G498) assisted threading**
T0950-D1	0.1494	0.1714	0.1635
T0951-D1	0.9338	0.9697	0.9697
T0953s1-D1	0.1748	0.2973	0.2055
T0953s2-D1	0	0.1569	0.1569
T0953s2-D2	0.0976	0.2841	0.2841
T0953s2-D3	0.1164	0.1477	0.1477
T0954-D1	0.3057	0.5741	0.5669
T0955-D1	0.1605	0.2099	0.2099
T0957s1-D1	0.1879	0.1879	0.1633
T0957s1-D2	0.3146	0.3146	0.2694
T0957s2-D1	0.2549	0.2792	0.2792
T0958-D1	0.3646	0.4019	0.4019
T0960-D1	0.1556	0.1501	0.1501
T0960-D2	0.1511	0.2289	0.2289
T0960-D3	0.1309	0.333	0.333
T0960-D4	0.1752	0.1264	0.1264
T0960-D5	0.1696	0.2121	0.2121
T0963-D1	0.1466	0.1726	0.1726

T0963-D2	0.1681	0.2646	0.2646
T0963-D3	0.1423	0.2212	0.2212
T0963-D4	0.1308	0.1178	0.1178
T0963-D5	0.1419	0.1398	0.1398
T0966-D1	0.8081	0.8081	0.8081
T0968s1-D1	0.2104	0.2847	0.2847
T0968s2-D1	0.2041	0.2657	0.2657
T1003-D1	0.8219	0.9033	0.9033
T1005-D1	0.5692	0.5692	0.5692
T1008-D1	0.389	0.389	0.389
T1009-D1	0.8074	0.8074	0.8074
T1011-D1	0.714	0.714	0.714
T1011-D2	0.9816	0.9816	0.9816
T1016-D1	0.802	0.8724	0.8724
**Mean**	**0.34**	**0.392**	**0.387**
**P-value**	-	0.0002	0.0008

For T0953s2-D1 target, pure threading predicts a model, which does not have any common residue to the native.

Supplementary Table S5. *p*-value of different contact-assisted threading methods on PSICOV150 dataset compared to baseline pure threading method. (*p*-values < 0.05 listed in bold)*

	mfDCA-assisted threading	PSICOV-assisted threading	MetaPSICOV-assisted threading
pure threading	**5.80E-08**	**2.90E-05**	0.07

* excluding two targets (1tqhA and 1hdoA) for which RaptorX could not predict contact maps.
